# The transcription factor CHOP, an effector of the integrated stress response, is required for host sensitivity to the fungal intracellular pathogen *Histoplasma capsulatum*

**DOI:** 10.1371/journal.ppat.1006589

**Published:** 2017-09-27

**Authors:** Bevin C. English, Nancy Van Prooyen, Tiit Örd, Tõnis Örd, Anita Sil

**Affiliations:** 1 Department of Microbiology and Immunology, University of California San Francisco, San Francisco, California, United States of America; 2 FLX Bio, South San Francisco, California, United States of America; 3 Institute of Molecular and Cell Biology, University of Tartu, Tartu, Estonia; 4 Estonian Biocentre, Tartu, Estonia; Geisel School of Medicine at Dartmouth, UNITED STATES

## Abstract

The ability of intracellular pathogens to manipulate host-cell viability is critical to successful infection. Some pathogens promote host-cell survival to protect their replicative niche, whereas others trigger host-cell death to facilitate release and dissemination of the pathogen after intracellular replication has occurred. We previously showed that the intracellular fungal pathogen *Histoplasma capsulatum* (*Hc*) uses the secreted protein Cbp1 to actively induce apoptosis in macrophages; interestingly, *cbp1* mutant strains are unable to kill macrophages and display severely reduced virulence in the mouse model of *Hc* infection. To elucidate the mechanism of Cbp1-induced host-cell death, we performed a comprehensive alanine scanning mutagenesis and identified all amino acid residues that are required for Cbp1 to trigger macrophage lysis. Here we demonstrate that *Hc* strains expressing lytic *CBP1* alleles activate the integrated stress response (ISR) in infected macrophages, as indicated by an increase in eIF2α phosphorylation as well as induction of the transcription factor *CHOP* and the pseudokinase *Tribbles 3* (*TRIB3*). In contrast, strains bearing a non-lytic allele of *CBP1* fail to activate the ISR, whereas a partially lytic *CBP1* allele triggers intermediate levels of activation. We further show that macrophages deficient for *CHOP* or *TRIB3* are partially resistant to lysis during *Hc* infection, indicating that the ISR is critical for susceptibility to *Hc*-mediated cell death. Moreover, we show that CHOP-dependent macrophage lysis is critical for efficient spread of *Hc* infection to other macrophages. Notably, *CHOP* knockout mice display reduced macrophage apoptosis and diminished fungal burden and are markedly resistant to *Hc* infection. Together, these data indicate that Cbp1 is required for *Hc* to induce the ISR and mediate a CHOP-dependent virulence pathway in the host.

## Author summary

*Histoplasma capsulatum* is the causative agent of histoplasmosis, a fungal infection that can be fatal in a wide range of mammalian hosts, including otherwise healthy, immunocompetent individuals. *Histoplasma* cells replicate to very high levels within host macrophages, eventually causing macrophage death and the release of live fungal cells. Here, we show that *Histoplasma* yeast use the secreted protein Cbp1 to activate a specific signaling pathway in the host cell to cause macrophage death during infection. Importantly, this signaling cascade is essential for pathogenesis, and mice deficient for a central component of this pathway are resistant to *Histoplasma* infection. Our study is the first demonstration that *Histoplasma* employs a secreted effector to alter host signaling pathways to promote virulence, and thus provides key insight into the pathogenesis strategies of this important human fungal pathogen.

## Introduction

Death of the host cell has profound consequences for an intracellular pathogen. It may eliminate the pathogen’s replicative niche, or it may promote pathogen spread. The specific form of cell death can impact immune activation of the host: pyroptosis and necroptosis are highly inflammatory types of cell death whereas apoptosis is generally characterized as immunologically quiet, although it does promote cross-presentation of antigens by dendritic cells. Thus, it is unsurprising that many intracellular pathogens have evolved various mechanisms and strategies to manipulate host-cell survival and death [1,2]. For example, *Mycobacterium tuberculosis* has been shown to inhibit apoptosis in macrophages to promote bacterial survival and replication, but also promote necrosis, allowing for bacterial spread [3].

The intracellular fungal pathogen *Histoplasma capsulatum* (*Hc*) is adept at manipulating the viability of macrophages during infection. *Hc* causes respiratory and systemic disease in a wide range of mammalian hosts, including immunocompetent individuals. *Hc* is a thermally dimorphic fungus, growing in the soil at ambient temperatures as a saprophytic mold that produces spores. When the soil is disturbed, spores and hyphae aerosolize and can be inhaled by mammalian hosts. After inhalation, exposure to mammalian body temperature is sufficient to induce a morphogenetic switch of the fungus, resulting in unicellular yeast-form growth. *Hc* yeast survive and replicate within host macrophages, eventually triggering host-cell death and allowing the release of live yeast cells.

*Hc*-dependent macrophage lysis is dependent on Cbp1, a secreted virulence factor of *Hc* that is produced specifically by the yeast phase of *Hc* [4]. In fact, Cbp1 is the most abundant secreted protein present in *Hc* culture supernatants [4], and it is critical for robust macrophage death and *in vivo* pathogenesis [5,6]. We previously demonstrated that Cbp1 is dispensable for high fungal burden but required for host-cell death, suggesting that it actively promotes macrophage death during infection. Indeed, during *Hc* infection, caspase-3/7 activation and host-cell death is fully dependent on Cbp1 and partially dependent on the pro-apoptotic host proteins Bax and Bak, suggesting that Cbp1 promotes apoptosis in *Hc*-infected macrophages [6].

Interestingly, Cbp1 has no identifiable protein domains and few identified homologs [7], and the mechanism by which it induces host-cell death is unknown. To generate tools to explore the host pathways that are impacted by Cbp1, we performed an alanine-scanning mutagenesis of Cbp1 and identified point mutants that were unable to lyse macrophages during *Hc* infection. These alleles were used to explore the mechanism by which *Hc* induces apoptosis in a Cbp1-dependent manner. We determined that Cbp1 activates the integrated stress response (ISR), which is one of several pathways that can trigger apoptosis in mammalian cells. The ISR is an intracellular signaling cascade that is activated after exposure to a variety of stresses, including endoplasmic reticulum (ER) stress and amino acid starvation. The central signaling event is the phosphorylation of the α-subunit of the eukaryotic initiation factor eIF2, which leads to a global reduction in translation levels. Despite this general translational inhibition, the translation of several key transcripts is promoted, resulting in a stress response that initially facilitates return to homeostasis but leads to apoptosis if the stress is unresolved. Here we demonstrate that *Hc* strains expressing lytic *CBP1* alleles trigger the integrated stress response (ISR) in macrophages during infection, leading to an induction of the pro-apoptotic genes *CHOP* and *Tribbles 3* (*TRIB3*). Non-lytic *CBP1* alleles are unable to induce *CHOP* and *TRIB3*. We further show that these components of the ISR are necessary for robust macrophage death during *in vitro* infection. Finally, we show that the fungal burden of *Hc* is significantly reduced in *CHOP* knockout mice, which are highly resistant to *Hc* infection. Together, these data demonstrate that *Hc* uses the secreted protein Cbp1 to activate the ISR in macrophages, resulting in full virulence of the pathogen in *in vitro* and *in vivo* infection models.

## Results

### Generation of *CBP1* mutant alleles with compromised ability to trigger macrophage lysis

**Fig 1:**
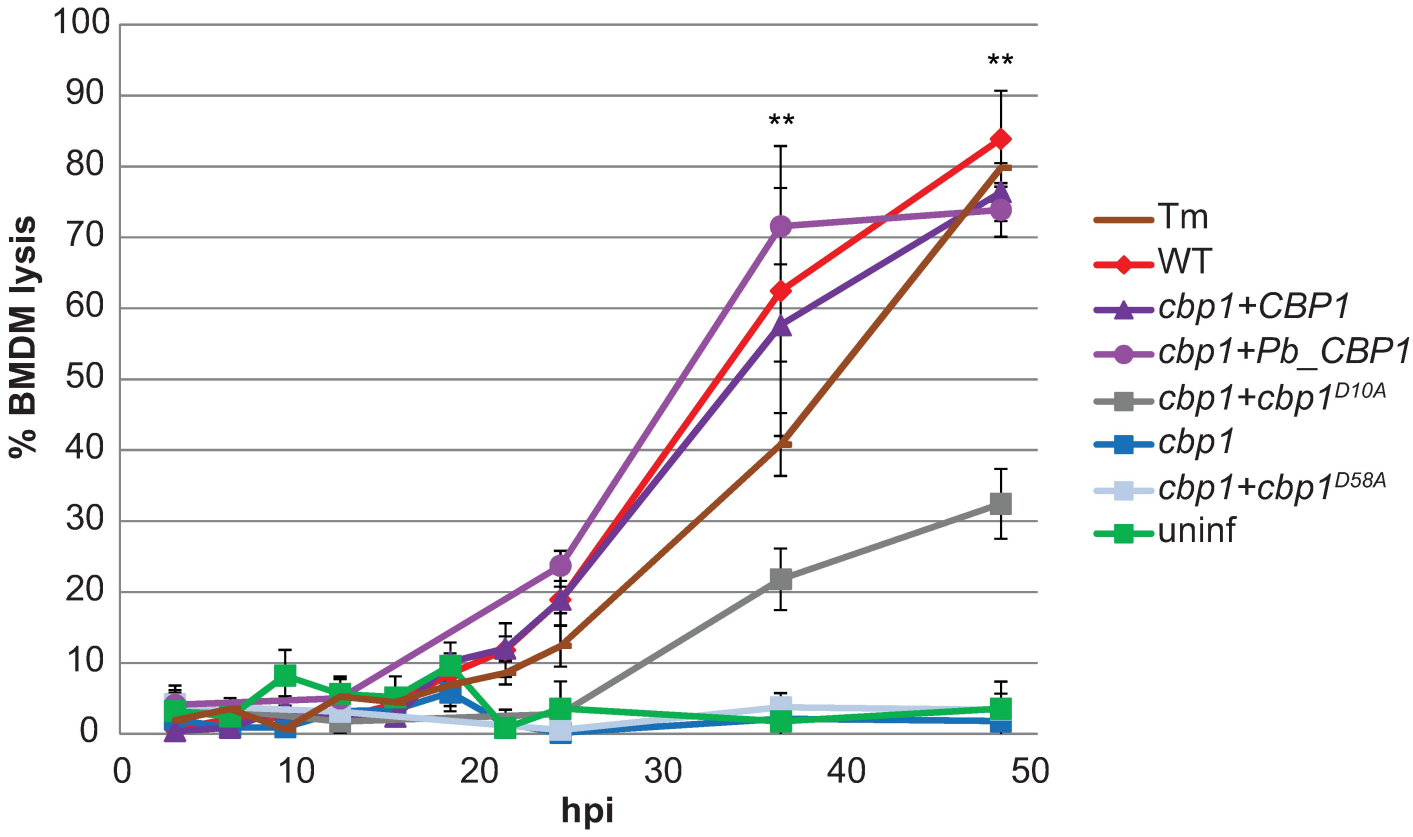
*CBP1* alleles show a range of lytic abilities. BMDMs were treated with 2.5 μg/mL tunicamycin (Tm), infected with indicated *Hc* strains at an MOI of 5, or mock infected (uninf). Macrophage lysis was measured by lactate dehydrogenase (LDH) release into culture supernatants and is presented as the percentage of total LDH in the supernatant and lysate of uninfected macrophages at 3 hpi. The lysis at each time point is an average of duplicate measurements of wells infected in triplicate, resulting in six total measurements, ± standard deviation. Macrophages treated with Tm or infected with wildtype *Hc*, *cbp1+CBP1*, *cbp1+Pb_CBP1*, or *cbp1+cbp1*^*D10A*^ showed significantly more lysis compared to uninfected BMDMs at the indicated time points (**p<0.01, two-tailed Mann-Whitney test).

To generate alleles of Cbp1 that perturb the ability of *Hc* to lyse macrophages, we conducted alanine scanning mutagenesis on Cbp1 in the highly virulent G217B strain background. Each of the 63 non-alanine amino acids in the 78 amino acid Cbp1 sequence were individually changed to alanine, and the resultant mutant *CBP1* alleles were expressed in the *cbp1* mutant strain. The resulting strains were then screened to determine which mutant proteins were present at high levels in yeast culture supernatants. Of the 63 mutant proteins, 20 were not detected in yeast culture supernatants (S1 Table; S1 Fig). We next examined whether the 43 secreted mutant proteins were able to rescue the defect in macrophage lysis seen with our *cbp1* mutant strain. To do so, we used the corresponding 43 strains to infect J774.1 cells, a murine macrophage-like cell line whose lysis during *Hc* infection is dependent on Cbp1 [6]. Mammalian cell death was qualitatively assessed by fixing and visualizing the monolayer with methylene blue (S1 Fig). Of the secreted mutants, 26 alanine mutants showed a reduction in macrophage lysis (S1 Table). The lysis defects of two of these secreted mutants, D10A and D58A (S2A Fig), were validated quantitatively using infected murine bone marrow-derived macrophages (BMDMs). The D10A mutant was able to partially complement the macrophage lysis defect of the *cbp1* strain, whereas D58A was completely defective for macrophage lysis (Fig 1). Importantly, the reduction in macrophage lysis after infection with the D10A mutant was not due to any differences in fungal burden; this mutant showed the same intracellular growth rate as wildtype *Hc* (S2B Fig). In contrast, the non-lytic D58A mutant showed reduced growth kinetics in macrophages (S2B Fig).

In addition to generating an allelic series of *CBP1* mutants by mutagenesis, we also examined the function of the *CBP1* homolog from the closely related fungus *Paracoccidioides brasiliensis*, another thermally dimorphic intracellular pathogen. *P*. *brasiliensis*, which is also able to colonize macrophages [8], is one of the few fungi with a clear ortholog of *Hc* Cbp1, although the function of the *Pb* protein is completely uncharacterized. *Pb* Cbp1 differs from *Hc* Cbp1 at 32 of its 77 amino acids (S3 Fig). Based on our perusal of general transcriptomics and proteomics studies in *Pb*, the *CBP1* transcript is highly expressed in *P*. *brasiliensis* yeast cultures compared to mycelia [9], and the protein is highly abundant in yeast culture supernatants [10]. We expressed a fusion protein consisting of the G217B Cbp1 signal peptide followed by the predicted mature Cbp1 from the Pb03 *P*. *brasiliensis* strain in our *Hc cbp1* mutant strain. After confirming that the protein (Pb_Cbp1) was secreted into *Hc* culture supernatants (S2A Fig), we then determined that it was able to fully rescue the lysis defect of the *Hc cbp1* mutant (Fig 1), with no change in intracellular growth kinetics compared to wildtype *Hc* (S2B Fig). We decided to use the wild-type *Hc* Cbp1, the nonlytic D58A mutant, the partially lytic D10A mutant, and the fully lytic Pb_Cbp1 strains to elucidate how Cbp1 causes macrophage death during *Hc* infection.

### *Histoplasma* does not cause ER stress in infected macrophages

**Fig 2:**
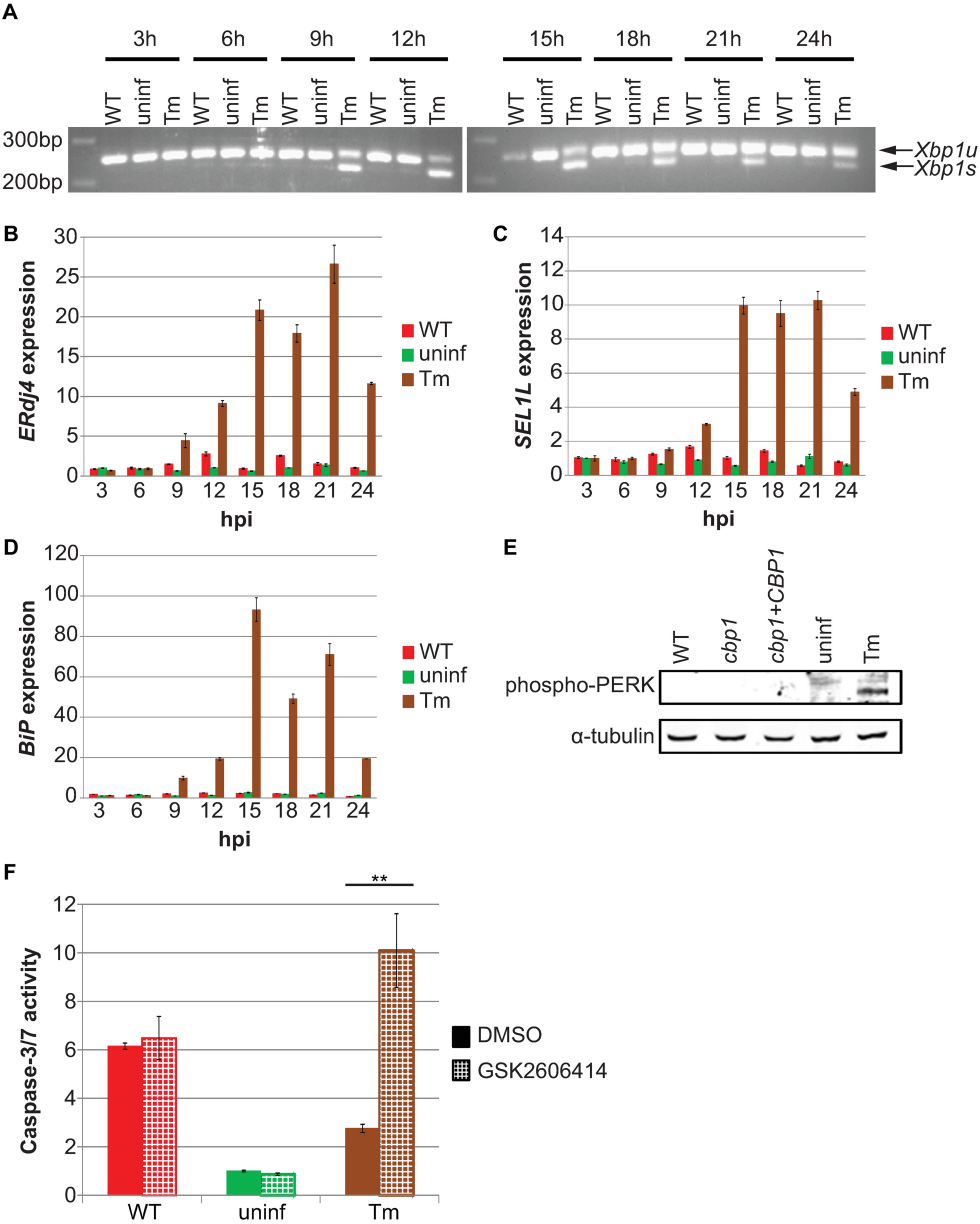
*Hc* does not cause ER stress in infected BMDMs. BMDMs were infected with the indicated *Hc* strains at an MOI of 5, mock infected (uninf), or treated with 2.5 μg/mL tunicamycin (Tm). **(A)** Unspliced (*Xbp1u*) and spliced (*Xbp1s*) isoforms of *Xbp1* were detected by non-quantitative RT-PCR. **(B-D)** Relative abundances of **(B)** *ERdj4*, **(C)** *SEL1L*, and **(D)** *BiP* were measured by RT-qPCR. **(E)** Phosphorylated PERK (Thr 980) was detected by Western blot at 12 hpi. **(F)** BMDMs were treated with the PERK-specific inhibitor GSK2606414 (3 μM) or vehicle (DMSO) for 12 hpi, and caspase-3/7 activity was assessed and normalized to vehicle-treated uninfected cells. Each value is an average of triplicate wells ± standard deviation. **p<0.01, two-tailed t test.

We previously identified a set of host genes that are upregulated in *Hc*-infected macrophages in a Cbp1-dependent manner, and many of these same genes are induced during ER stress [6]. This finding was intriguing for two reasons: many intracellular pathogens manipulate ER stress pathways in the host [11], and unresolved ER stress can lead to apoptosis through many mechanisms [12]. To determine if *Hc* triggers ER stress in macrophages, we assessed the activation of the unfolded protein response (UPR) during infection. The mammalian UPR, which counters the effects of ER stress, consists of three distinct signaling pathways triggered by three ER-resident proteins that are able to detect ER stress: IRE1, ATF6, and PERK [12–14] (S4 Fig). Activated IRE1 is an RNase which removes a non-canonical intron in the transcript encoding the transcription factor Xbp1 [15]. To examine IRE1 activation, we compared levels of unspliced and spliced Xbp1 transcripts (*Xbp1u* and *Xbp1s*, respectively) in macrophages infected with *Hc* or treated with the potent ER stress-inducer tunicamycin (Tm), which activates all three branches of the UPR. Infection with *Hc* did not result in an increase in *Xbp1s* compared to uninfected macrophages, whereas *Xbp1s* was detected in macrophages as early as 9 hours after Tm treatment (Fig 2A). To further assess IRE1 activation, we also examined the induction of the Xbp1 target gene *ERdj4* [16]. Similar to *Xbp1s*, *ERdj4* was upregulated 9 hours after the addition of Tm but was not upregulated in *Hc*-infected macrophages (Fig 2B). We then examined ATF6, which is cleaved during ER stress, resulting in the transcription factor ATF6n. To assess the activation of ATF6, we monitored the upregulation of one of its specific targets, *SEL1L* [17]. Similar to *ERdj4*, *SEL1L* was induced by Tm but not induced in *Hc*-infected macrophages (Fig 2C). We also examined the transcript levels of the chaperone protein *BiP*/*GRP78*, a classic marker of ER stress whose upregulation is dependent on both IRE1 and ATF6 [18]. *BiP* was strongly induced after Tm treatment, but was not upregulated in macrophages after *Hc* infection (Fig 2D). Next, we looked at the third and final mammalian UPR sensor, PERK, which oligomerizes and autophosphorylates upon activation [19,20]. We were unable to detect phospho-PERK in *Hc*-infected macrophages (Fig 2E), suggesting that *Hc* infection does not activate PERK. To confirm this hypothesis, we utilized the PERK-specific inhibitor GSK2606414 [21]. Consistent with previous observations [22], inhibition of PERK signaling increased caspase-3/7 activity in cells after tunicamycin treatment. However, GSK2606414 had no effect on caspase-3/7 activity in *Hc*-infected macrophages (Fig 2F), consistent with our observation that PERK is not activated during *Hc* infection. Taken together, these data demonstrate that *Hc* does not activate the UPR in infected macrophages.

### *Hc* strains expressing lytic *CBP1* alleles activate the integrated stress response (ISR) in macrophages

**Fig 3:**
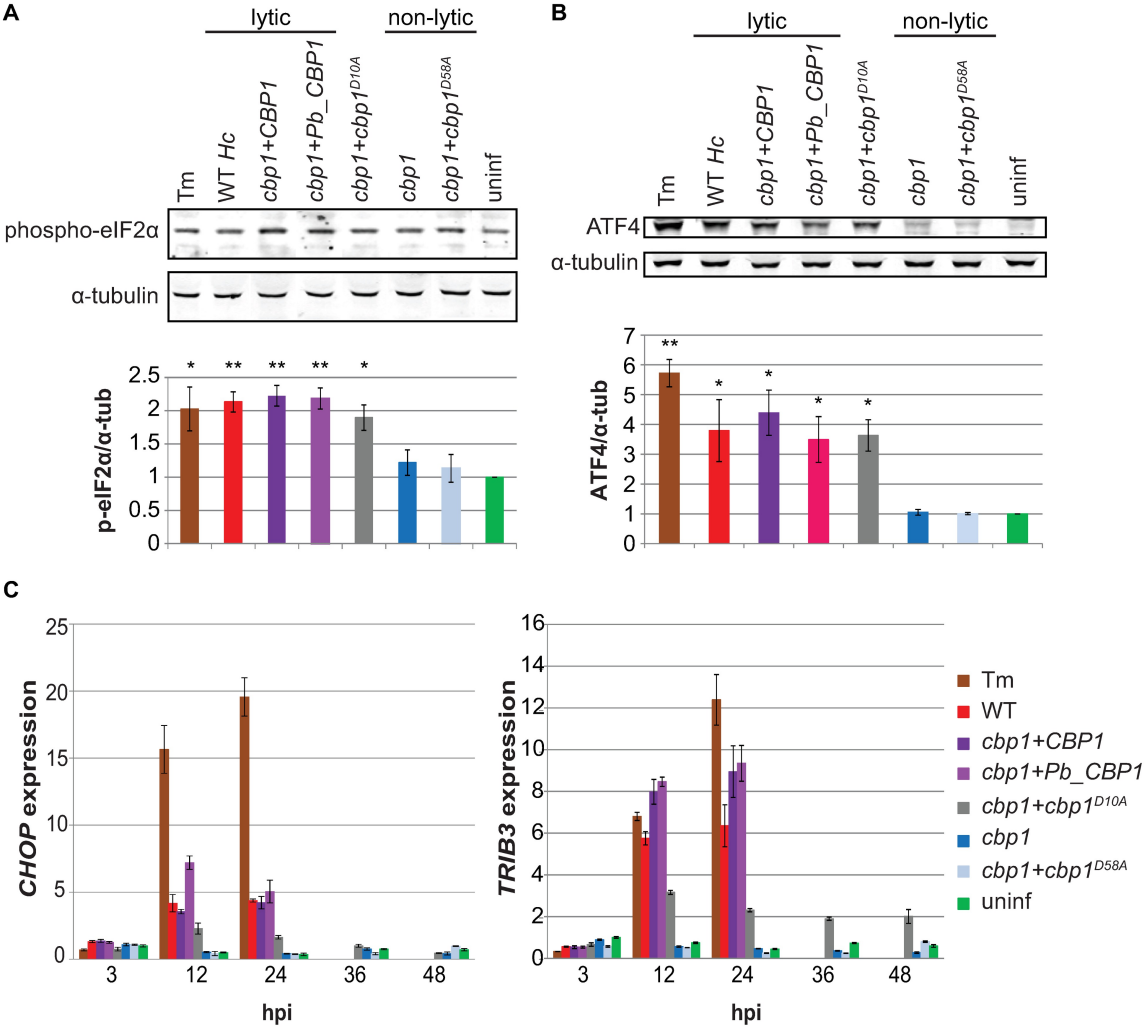
Lytic *CBP1* alleles activate the integrated stress response in infected macrophages. BMDMs were treated with 2.5 μg/mL tunicamycin (Tm), infected with indicated *Hc* strains at an MOI of 5, or mock infected (uninf). Phosphorylated eIF2α (Ser51) **(A)** and ATF4 **(B)** were assessed by Western blot at 12 hpi. Representative blots are shown. The average signal intensity of phospho-eIF2α or ATF4 relative to the corresponding α-tubulin loading control from three replicates is shown in the bar graphs.*p<0.05, **p<0.01, compared to wildtype, two-tailed t-test. **(C)** Relative abundances of *CHOP* and *TRIB3* transcripts were assessed by RT-qPCR at the indicated time points and normalized to uninfected BMDMs at 3 hpi.

After determining that *Hc*-infected macrophages are not undergoing ER stress, we then re-examined our transcriptional profiling results [6]. Many of the genes that are upregulated during infection in a Cbp1-dependent manner are involved in the integrated stress response (ISR), a pathway activated in response to a variety of cellular stresses, including ER stress. Thus, we shifted our focus to the ISR. The central event in the ISR signaling cascade is the phosphorylation of the α-subunit of the translation initiation factor eIF2 [23,24]. Phosphorylation of eIF2α leads to an overall reduction in translation, but allows for the increased translation of select, stress-responsive transcripts due to unique features in their mRNAs. To first examine ISR activation, we assessed phospho-eIF2α levels in macrophages by Western blot. We observed an increase in phospho-eIF2α in macrophages infected with *Hc* strains expressing lytic alleles of *CBP1*. Though it was a modest increase in phosphorylated eIF2α levels in macrophages infected with lytic *Hc* strains, it was comparable to the increase seen in cells treated with Tm (Fig 3A), and small changes in phospho-eIF2α levels are known to have large effects in the cell [25]. Importantly, we observed this increase in phospho-eIF2α at 12 hours post-infection (hpi), which is well before the onset of macrophage lysis (Fig 1). To confirm ISR activation, we next looked at the levels of ATF4, a transcription factor that is preferentially translated as phospho-eIF2α levels increase [26,27]. As expected, we saw robust levels of ATF4 in macrophages infected with *Hc* expressing lytic alleles of *CBP1*. ATF4 induction preceded the onset of macrophage lysis and correlated with phospho-eIF2α levels (Fig 3B).

To continue our exploration of the ISR, we also examined the expression of two downstream targets of ATF4, the transcription factor *CHOP/DDIT3* and pseudokinase *TRIB3* [28], which were previously implicated by our transcriptional analysis of infected cells [6]. Upregulation of CHOP and TRIB3, a target of CHOP, has been shown to be pro-apoptotic in a variety of cell types in response to multiple stresses [29–33]. Both *CHOP* and *TRIB3* showed robust induction in macrophages after infection with lytic *Hc* strains (Fig 3C), and this correlated with protein levels (S5 Fig). Notably, there was intermediate induction in macrophages infected with *Hc* expressing the partially lytic D10A *CBP1* alanine mutant (Fig 3C). Furthermore, robust *TRIB3* expression after infection with *Hc* secreting wildtype Cbp1 is dependent on *CHOP*, as *CHOP^-/-^* macrophages showed reduced *TRIB3* induction after infection with wildtype *Hc* or the complemented *cbp1* mutant strain (S6 Fig). Together, these data demonstrate that *Hc* triggers the ISR in macrophages before the onset of host-cell death, and the activation of the ISR is dependent on the expression of lytic *CBP1* alleles. Importantly, at 48 hpi, we did not see an increase in phospho-eIF2α, ATF4, *CHOP*, or *TRIB3* levels in macrophages infected with non-lytic *Hc* strains, even though the fungal burden of the non-lytic strains at this time point exceeds that of the lytic strains at 12 hpi (S2 Fig). Thus, a high intracellular fungal burden is not sufficient to activate the ISR in infected macrophages.

To determine if ISR activation was a general response of different macrophage types to divergent *Histoplasma* strains, we varied either the *Hc* strain or the host macrophage and assessed ISR acviation by examining *CHOP* and *TRIB3* induction. First, we infected BMDMs with G186AR, a clinical isolate from a different *Histoplasma* clade than G217B [34]. We observed robust *CHOP* and *TRIB3* induction in infected BMDMs before the onset of macrophage death (S7A Fig). Next, we used the G217B strain to infect U937 cells, a human cell line that can be differentiated to a macrophage-like state. Similar to what we observed with BMDMs, these human cells displayed Cbp1-dependent lysis as well as Cbp1-dependent *CHOP* and *TRIB3* induction during *Hc* infection (S7B Fig). Together, these data indicate that ISR activation in macrophages during *Hc* infection occurs in multiple biological contexts.

### A reduction in phospho-Akt precedes host-cell death in macrophages infected with *Hc* strains expressing lytic *CBP1* alleles

**Fig 4:**
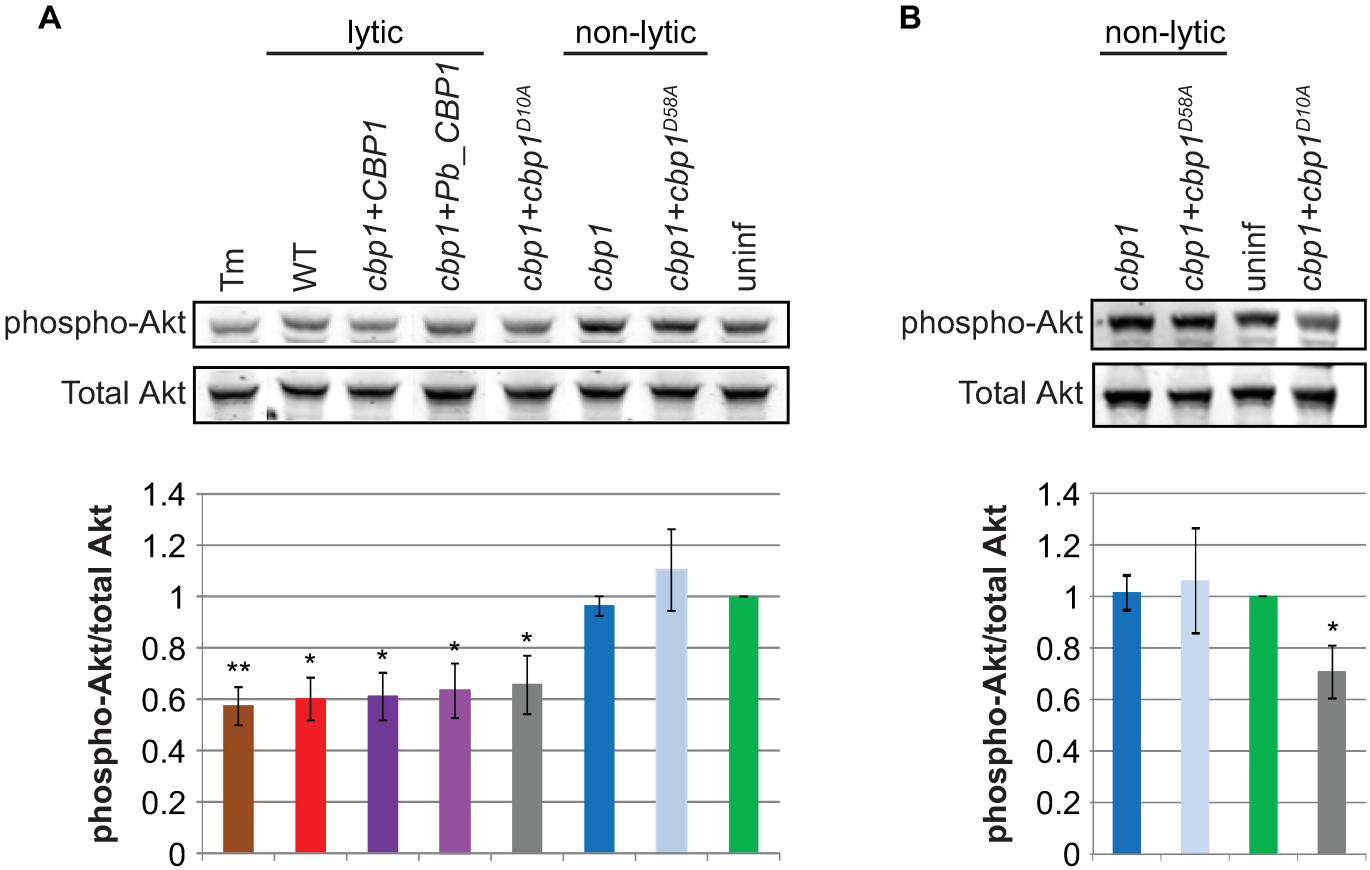
Lytic *CBP1* alleles cause a decrease in phospho-Akt levels in infected macrophages. BMDMs were infected with the indicated *Hc* strains, and phospho-Akt (Thr308) and total Akt were assessed by Western blot at **(A)** 12 hpi and **(B)** 48 hpi. Representative blots are shown. The average signal intensity of phospho-Akt relative to total Akt from three replicates is shown in the bar graphs.*p<0.05, **p<0.01, compared to wildtype, two-tailed t-test.

The pro-apoptotic pseudokinase Trib3 is hypothesized to promote apoptosis by binding Akt and preventing its phosphorylation, thereby reducing its pro-survival and anti-apoptotic signaling [35]. After determining that *TRIB3* is induced during *Hc* infection, we then evaluated phospho-Akt levels in infected macrophages. Macrophages infected with lytic *Hc* strains showed a reduction in phospho-Akt levels at 12 hpi, whereas macrophages infected with non-lytic *Hc* strains showed no such reduction (Fig 4A). Importantly, those macrophages infected with non-lytic *Hc* strains showed no reduction in phospho-Akt, even 48 hpi (Fig 4B), when the fungal burdens of the non-lytic strains exceed those of the lytic strains at 12 hpi (S2 Fig).

### Caspase-8 is activated in macrophages infected with *Histoplasma* in a Cbp1-dependent manner

**Fig 5:**
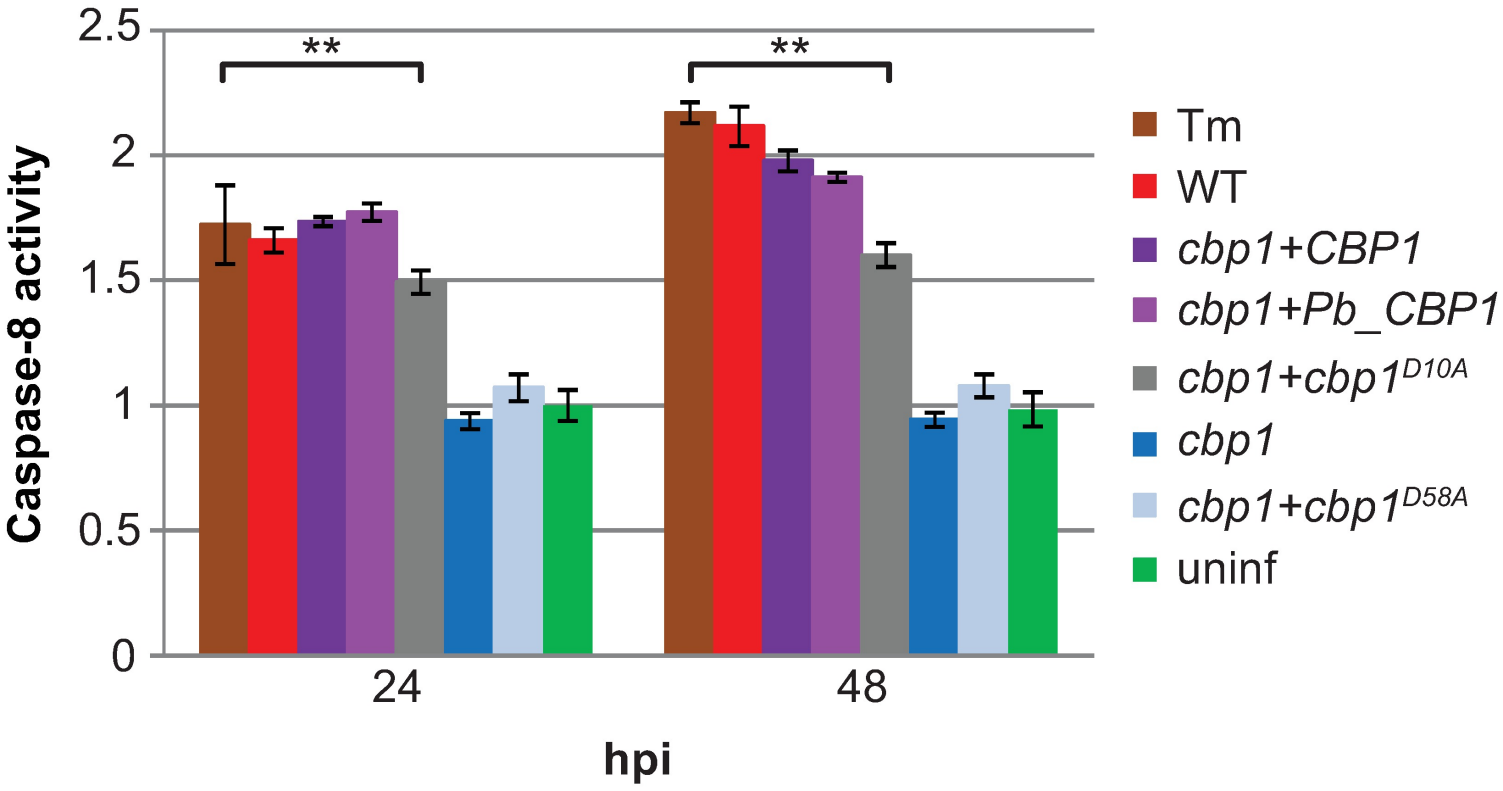
Lytic *CBP1* alleles cause an increase in caspase-8 activity in infected macrophages. BMDMs were treated with 2.5μg/mL tunicamycin (Tm), infected with indicated *Hc* strains at an MOI of 5, or mock infected (uninf). Caspase-8 activity was measured at 24 and 48 hpi and normalized to the activity in uninfected cells at 24 hpi. Each value is an average of triplicate wells ± standard deviation. **p<0.005, compared to wildtype, two-tailed t-test.

In addition to reducing phospho-Akt levels, ISR signaling has been shown to promote apoptosis by activating caspase-8 [36]. Caspase-8 is an initiator caspase that is able to directly activate downstream caspases, thereby bypassing the need for mitochondrial outer membrane permeabilization (MOMP), canonically considered the central signaling event in the intrinsic apoptosis pathway [37]. Macrophages infected with lytic *Hc* strains showed an increase in caspase-8 activity that was comparable to that of macrophages treated with tunicamycin. In contrast, macrophages infected with non-lytic *Hc* strains showed no increase in caspase-8 activity when compared to uninfected macrophages (Fig 5). Thus, expression of functional Cbp1 led to caspase-8 activation in macrophages during *Hc* infection.

### *CHOP* and *TRIB3* are necessary for robust macrophage lysis during *Hc* infection

**Fig 6:**
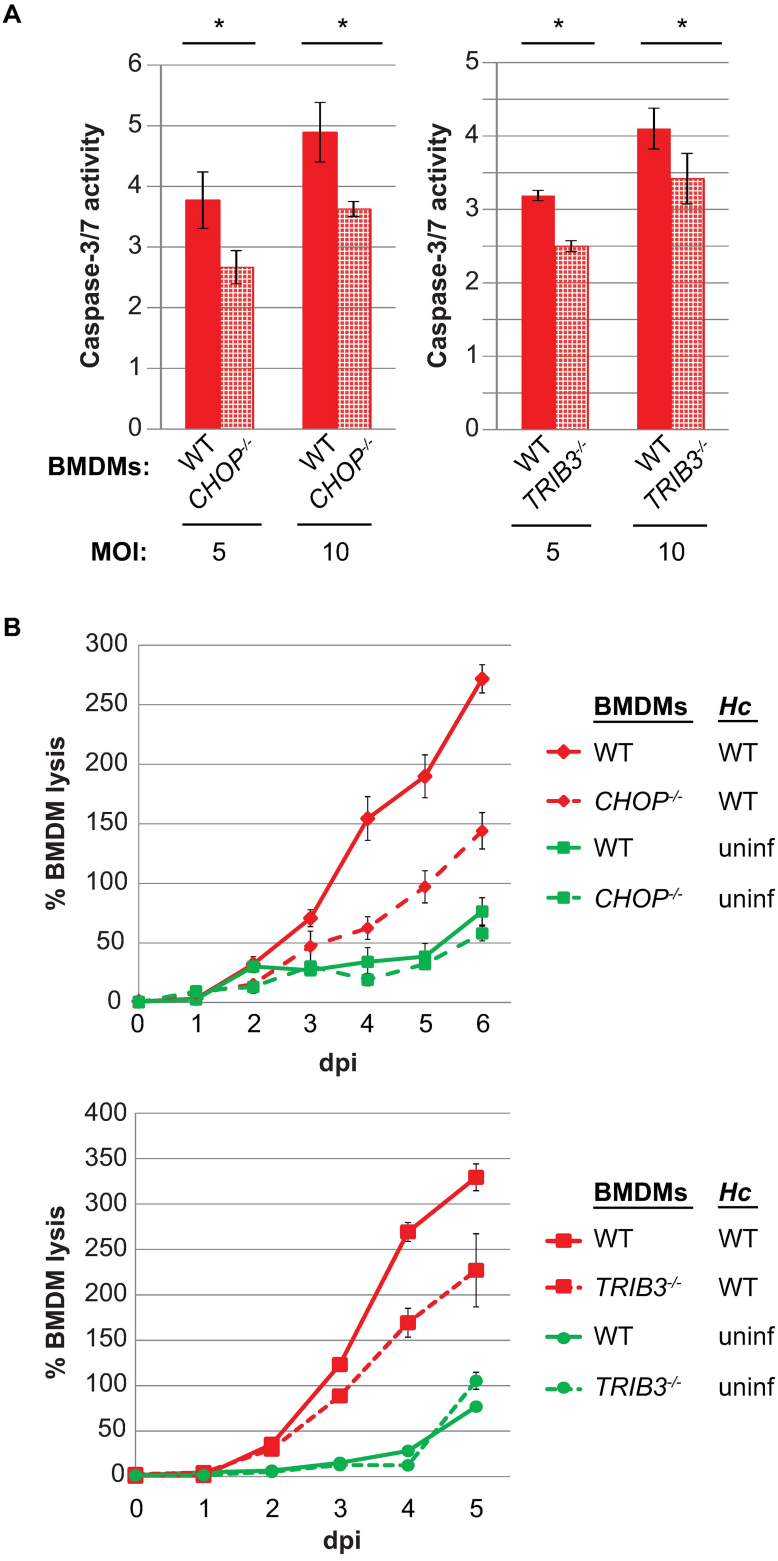
The host genes *CHOP* and *TRIB3* are necessary for robust caspase-3/7 activity and macrophage death during *Hc* infection. **(A)** Wildtype, *CHOP*^-/-^, and *TRIB3*^-/-^ macrophages were mock infected or infected with wildtype *Hc* at an MOI of 5 or 10. Caspase-3/7 activity was measured 24 hpi and normalized to the activity in uninfected macrophages for each genotype. Each value is an average of triplicate wells ± standard deviation. *p<0.05, compared to wildtype, two-tailed t-test. **(B)** Wildtype, *CHOP*^-/-^, and *TRIB3*^-/-^ BMDMs (MΦ) were infected with wildtype *Hc* at an MOI of 1 or mock infected (uninf), and macrophage lysis was measured by LDH release. BMDM lysis is presented as the percentage of total LDH in the supernatant and lysate of uninfected macrophages at 3 hpi. The lysis at each time point is an average of duplicate measurements of wells infected in triplicate, resulting in six total measurements, ± standard deviation.

Having concluded that the ISR was activated before the onset of lysis, we next wanted to assess the relevance of this signaling pathway during *in vitro* infection. To this end, we generated BMDMs from *CHOP*^-/-^ [33] and *TRIB3*^-/-^ [38] knockout mice. Both *CHOP*^-/-^ and *TRIB3*^-/-^ macrophages showed a reduction in caspase-3/7 activity after infection with wildtype *Hc* (Fig 6A). The reduction in the activity of these executioner caspases correlated with a reduction in host-cell lysis after infection with wildtype *Hc* (Fig 6B). Importantly, these reductions in macrophage death were not due to any differences in fungal burden (S8 Fig). Thus, macrophages individually deficient for two different components of the ISR showed a reduction in lysis, demonstrating that activation of the ISR by *Hc* is required to trigger robust host-cell death during infection.

### CHOP is required for maximal spread of *Hc* during macrophage infection

**Fig 7:**
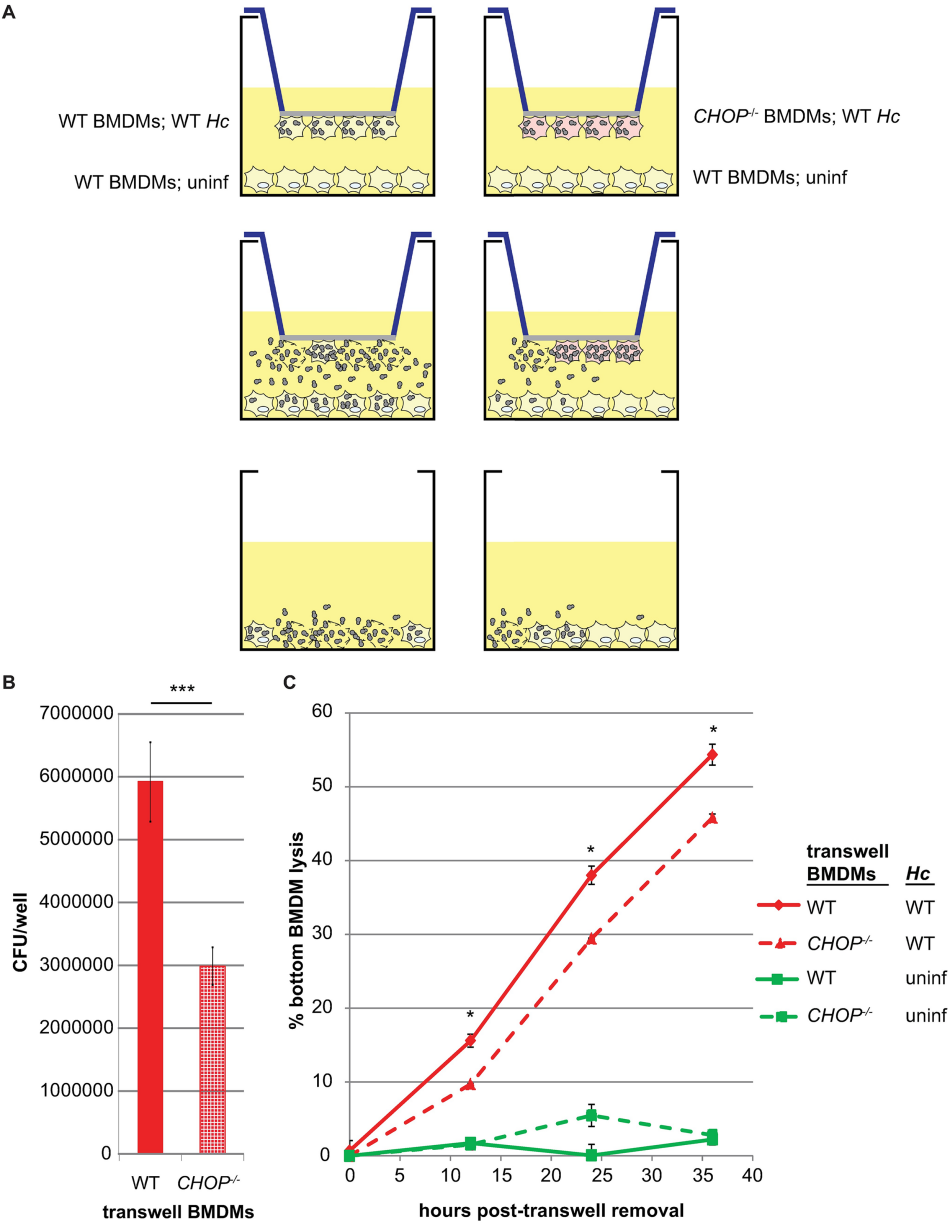
CHOP is required for optimal spread of *Hc* during macrophage infection. **(A)** Uninfected wildtype BMDMs were seeded in 6-well plates. Above them, transwells with wildtype or *CHOP*^*-/-*^ BMDMs, either uninfected or infected with wildtype *Hc*, were placed with the macrophages on the underside of the transwell. After the onset of lysis of the infected macrophages, the transwells were removed. The fungal burdens immediately after transwell removal **(B)** and lysis kinetics **(C)** of the bottom macrophages were assessed by CFU enumeration and LDH release assay, respectively. For CFUs, ***p<0.005 by two-tailed t-test; for macrophage lysis, *p<0.05 by two-tailed Mann-Whitney.

Intracellular pathogens can trigger host-cell death to facilitate efficient exit from a host cell, thereby promoting subsequent spread of infection to other host cells. Since optimal host-cell death during *Hc* infection is dependent on CHOP, we next tested whether CHOP affects *Hc* spread to other macrophages. We hypothesized that the reduction in lysis in *CHOP*^*-/-*^ macrophages would result in the release of fewer fungal cells, thereby reducing the spread of *Hc* to other macrophages. To test this hypothesis, we utilized the underside of transwells to seed wildtype or *CHOP*^*-/-*^ BMDMs that were mock infected or infected with wildtype *Hc*. These samples were then placed over uninfected, wildtype BMDMs so that these host cells were accessible to *Hc* released from the original wildtype or *CHOP*^*-/-*^ macrophages (Fig 7A). After the transwell macrophages had begun to lyse, but before the bottom macrophages lysed, we removed the transwells and assessed fungal burden in the lower macrophages. The bottom macrophages that had received *Hc* from infected *CHOP*^*-/-*^ BMDMs had a significantly lower fungal burden and reduced host-cell death compared to the bottom macrophages that had received *Hc* from wildtype BMDMs (Fig 7B and 7C). These data support the hypothesis that the reduced death of *CHOP*^*-/-*^ macrophages results in reduced spread of *Hc*, thereby limiting pathogenesis.

### *CHOP*^-/-^ mice are resistant to *Hc* infection

**Fig 8:**
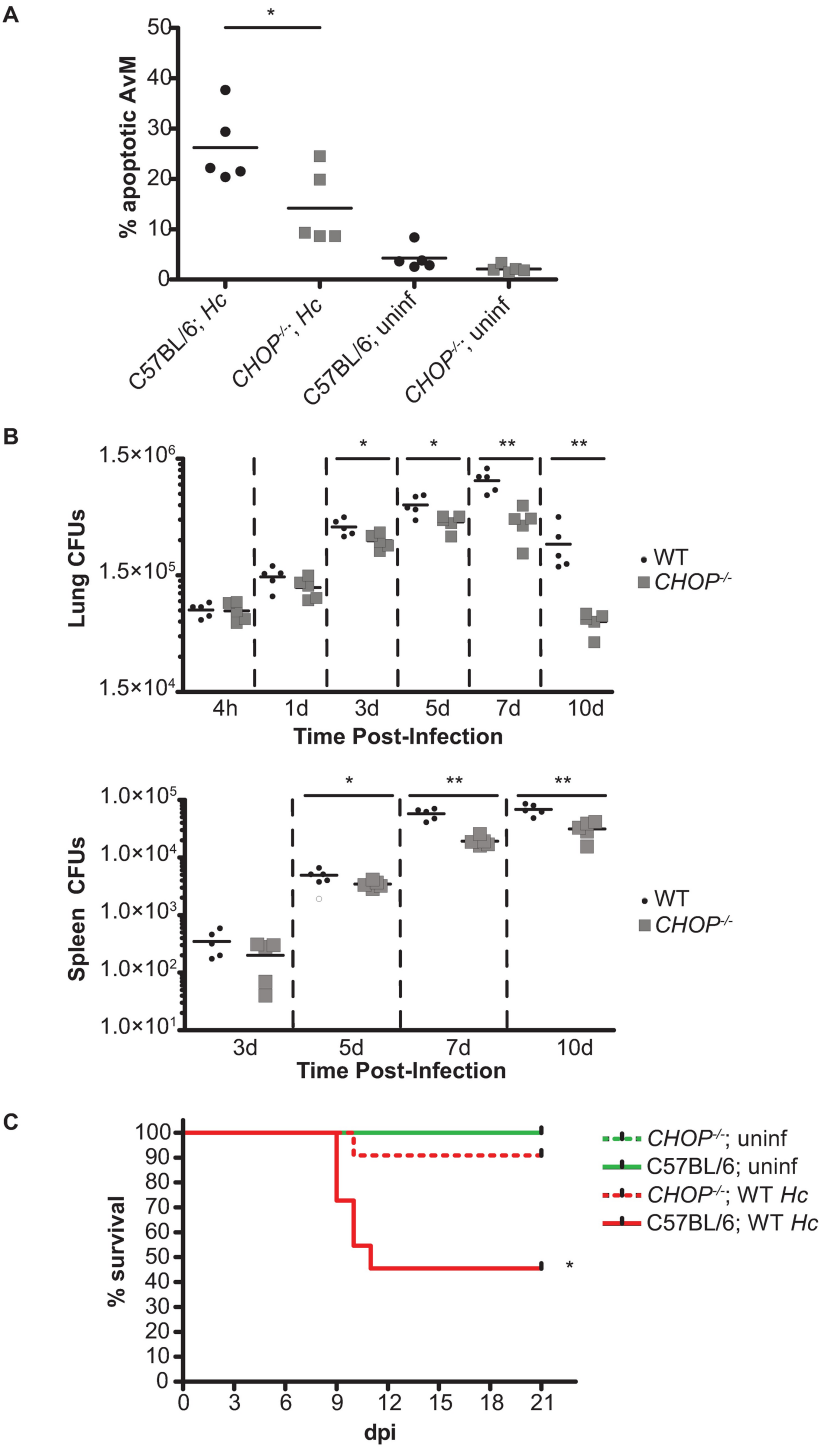
*CHOP*^*-/-*^ mice are resistant to *Hc* infection. **(A)** C57BL/6 and *CHOP*^*-/-*^ mice were mock infected (uninf) or infected with 1x10^6^ *Hc* yeast and the percentage of apoptotic (viability dye^+^ and caspase-3/7^+^) alveolar macrophages (CD45^+^ SiglecF^+^ CD11b^mid^ CD11c^+^ F4/80^+^) was measured at 3 dpi. *p<0.05, as determined by ANOVA. (B) CFUs from lungs and spleens of C57BL/6 and CHOP-/- mice infected with 3 x 10^5^ *Hc* yeast. *p<0.05, **p<0.005, as determined by independent 1-way ANOVA analyses of log transformed CFUs at each time point. (C) Kaplan-Meier survival curves of C57BL/6 and *CHOP*^-/-^ mice mock infected (uninf; n = 2) or infected with 1 x 10^6^ *Hc* yeast (n = 11). *p<0.05, logrank test.

Having determined that the ISR is necessary for full *Hc* virulence during *in vitro* infection, we next wanted to examine the relevance of the ISR during *in vivo* infection. We first asked if there was reduced macrophage apoptosis in *CHOP*^*-/-*^ mice following *Hc* infection. We infected *CHOP*^*-/-*^ and C57BL/6 mice intranasally with 1 x 10^6^ mCherry-producing *Hc* yeast [39] and performed flow cytometry analysis on lung homogenates at 3 dpi. There was no difference in the percentage of infected (i.e., mCherry^+^) cells in wild-type and *CHOP*^*-/-*^ animals at this time point (S9A Fig), although we were unable to determine the precise fungal burden per cell. Despite comparable numbers of infected cells, there was a significant reduction in the percentage of apoptotic alveolar macrophages in the lungs of infected *CHOP*^*-/-*^ animals compared to infected wild-type animals (Fig 8A).

We then tested whether this reduction in host-cell death correlated with a reduced fungal burden *in vivo*, especially in light of our observation that CHOP is required for efficient spread of *Hc in vitro* (Fig 7). We infected female *CHOP^-/-^* and C57BL/6 mice intranasally with a sub-lethal dose (3 x 10^5^ colony-forming units (CFU)/mouse) of wildtype *Hc* and then monitored fungal burden in the lungs and spleens of the infected animals. Even by 3 dpi, we observed a significant reduction in fungal burden in the lungs of *CHOP*^*-/-*^ mice compared to wildtype mice (Fig 8B). Similar to what we observed in the lungs, there was a significant reduction in *Hc* recovered from the spleens of *CHOP*^-/-^ mice compared to wildtype animals beginning at 5 dpi (Fig 8B). Importantly, this difference in fungal burden arises early during infection, before the activation of the adaptive immune response [39,40], consistent with our hypothesis that *CHOP* plays an important role in innate immune cells, such as macrophages, during *Hc* infection. Furthermore, as further evidence of ISR induction *in vivo*, we monitored *TRIB3* expression in the lungs of *CHOP*^-/-^ mice at 1 dpi, when there is no statistically significant difference in fungal burden between wildtype and mutant mice. Consistent with our *in vitro* observations, we observed that robust *TRIB3* induction *in vivo* is dependent on *CHOP* (S9B Fig; Fig 8B).

Finally, we then examined if the difference in fungal burden correlated with the outcome of infection. We infected female *CHOP*^-/-^ and C57BL/6 mice intranasally with a lethal dose (1 x 10^6^ CFU/mouse) of wildtype *Hc* and then monitored the animals daily for symptoms, including weight loss, hunching, panting, and lack of grooming. While all infected animals developed symptoms, including weight loss (S9C Fig), the wildtype mice (n = 11) showed more severe symptoms, with over half of the animals succumbing to infection. Notably, the *CHOP*^-/-^ mice were significantly more resistant to infection (Fig 8C), indicating that *CHOP* is required for virulence of *Hc in vivo*. Together, these data support the model that *Hc* activates the ISR in a *CHOP*-dependent manner, thereby promoting host-cell death and facilitating pathogen spread during infection.

## Discussion

*Hc* is a primary fungal pathogen that is able to replicate to very high levels within host macrophages before host-cell lysis occurs. However, as we previously demonstrated, high intracellular fungal burden is not sufficient to induce host-cell death. This conclusion is based on the phenotype of mutant strains that lack the small, secreted protein Cbp1: the *cbp1* mutant replicates to high levels within macrophages but fails to lyse them [6]. Here, we demonstrate that *Hc* strains expressing functional alleles of *CBP1* induce a host signaling pathway called the integrated stress response (ISR) in infected macrophages. Two pro-apoptotic components of ISR signaling, the transcription factor CHOP and the pseudokinase TRIB3, are necessary for robust macrophage death during *in vitro* infection. Furthermore, we show that CHOP is required for efficient spread of *Hc* during macrophage infection. Finally, in the mouse model of *Histoplasma* infection, CHOP is required for optimal macrophage apoptosis, fungal burden, and host sensitivity to *Hc*. The phrase “Integrated Stress Response” is a term that has been used for over a decade to describe a signaling cascade that is activated after mammalian cells undergo a variety of stresses, including ER stress, amino acid starvation, and iron deprivation [23,24,41]. The key event in this signaling pathway is the phosphorylation of the alpha subunit of the translation initiation factor eIF2. There are four known eIF2α kinases: general control non-depressible 2 (GCN2), which is activated by amino acid starvation; heme-regulated eIF2α kinase (HRI), which is activated during heme deprivation; protein kinase R (PKR), which is activated by cytosolic double-stranded RNA; and PKR-like ER kinase (PERK), which is activated by ER stress [42,43]. Phosphorylation of eIF2α by these kinases causes a reduction in global translation within the cell. Specific transcripts, however, are preferentially translated as phospho-eIF2α levels increase, resulting in a response geared to help the cell adapt to the stress. If the cell is unable to overcome the stress that initiated ISR signaling, the cascade ultimately leads to apoptosis of the cell through induction of many pro-apoptotic factors, including the transcription factor CHOP and its target, the pseudokinase Tribbles 3 [12].

The ability of the ISR to either combat stress or drive the cell towards apoptosis makes it a key target for intracellular pathogens. One well-studied mechanism of ISR manipulation by pathogens is alteration of ER stress signaling pathways. Different types of intracellular pathogens, ranging from viruses [44], to bacteria [11], to eukaryotic parasites [45,46], trigger the ISR in their host cells by inducing ER stress. Unsurprisingly, activation of the ISR has a range of outcomes, depending on the pathogen and the host cell. For example, the intracellular bacterium *M*. *tuberculosis* induces ER stress in macrophages, ultimately leading to apoptosis [47]. However, activation of this host signaling pathway is detrimental for the bacteria, as increasing phospho-eIF2α levels reduces bacterial burden, and similarly, reducing CHOP levels increases bacterial burden [48].

In the case of *Hc*, the ISR is triggered without any evidence of ER stress induction, and indeed it is clear that other microbial pathogens promote ISR activation via multiple pathways. For example, conditioned media from *Pseudomonas aeruginosa* cultures has been shown to induce ISR activation that is dependent on HRI. Additionally, *P*. *aeruginosa* also induces ER stress, although the functional outcome of these two ISR activating events during infection is unclear [49]. Reoviruses have been shown to trigger the ISR in host cells by activating PKR, an eIF2α kinase that directly senses cytosolic dsRNA [50,51]. This signaling benefits the pathogen, as attenuation of the ISR response by blocking eIF2α phosphorylation or ATF4 accumulation results in reduced viral replication [52]. In the case of *Hc*, the mechanism of Cbp1-dependent ISR induction is unknown. Since Cbp1 is a protein with no known functional domains, it is intriguing to consider exactly how this virulence factor triggers the ISR during *Hc* infection, and work to elucidate this mechanism is underway.

To advance a mechanistic understanding of Cbp1, we identified amino acids in Cbp1 that are critical for its ability to trigger host-cell lysis (S1 Table). These residues may affect Cbp1 protein structure or a potential ligand interaction site. Thus, the alanine mutant library generated here provides a significant tool for future structure-function studies. Additionally, we demonstrated that the presumptive Cbp1 ortholog from the closely related fungal pathogen *P*. *brasiliensis* (*Pb*) is able to fully complement the lysis defect of Cbp1-deficient *Hc*. To our knowledge, this is the first functional characterization of *Pb* Cbp1. It is intriguing to note that the sequences of *Hc* Cbp1 and *Pb* Cbp1 show many differences, with nearly half of the residues differing between G217B (*Hc*) and Pb03 (*Pb*) Cbp1 (S3 Fig). While our alanine scan results suggest that many of the N-terminal amino acid residues in *Hc* Cbp1 are necessary for macrophage death (S1 Table), *Pb* Cbp1 lacks these charged residues but is still able to induce robust host-cell lysis (S3 Fig). Additionally, and perhaps unsurprisingly, residues that are highly conserved between *Hc* Cbp1 and *Pb* Cbp1, such as D58, are essential for robust macrophage death. Interestingly, one of the few other fungal organisms that contains a Cbp1 homolog in its genome is *Emmonsia crescens* (S3 Fig). *Emmonsia* spp. are emerging dimorphic fungal pathogens [53], and the role of Cbp1 in their pathogenesis has yet to be explored. The acquisition of new sequence information in related fungi will enhance our ability to gain insight on structure-function analysis of Cbp1.

One of the highlights of the current study is the exploration of the role of CHOP in *Hc* infection, since the role of ISR signaling during *in vivo* infection with microbial pathogens is relatively unexplored. To our knowledge, only one study has previously determined the susceptibility of CHOP-deficient mice to bacterial infection: *CHOP*^*-/-*^ mice show reduced mortality in a polymicrobial sepsis model, and this increase in survival correlates with a decrease in bacterial burden [54]. Our data are the first to show that CHOP plays a role in host susceptibility to fungi, since we found that *CHOP*^*-/-*^ mice are resistant to *Hc* infection. Intriguingly, *Hc* was unable to achieve a robust increase in fungal burden in the absence of CHOP signaling in the host. This decreased fungal burden manifested in the lungs of *CHOP*^*-/-*^ mice by 3 dpi and in the spleen by 5 dpi. Importantly, the relatively early failure of *Hc* to thrive in the *CHOP*^*-/-*^ mice is consistent with an altered interaction between the fungus and innate immune cells such as alveolar and tissue macrophages. Based on our observations in cell culture and the reduced apoptosis in alveolar macrophages in infected *CHOP*^*-/-*^ mice, we speculate that *Hc* yeast are unable to robustly lyse macrophages in the *CHOP*^*-/-*^ mouse, thereby limiting the spread of the pathogen and the extent of fungal growth.

**Fig 9:**
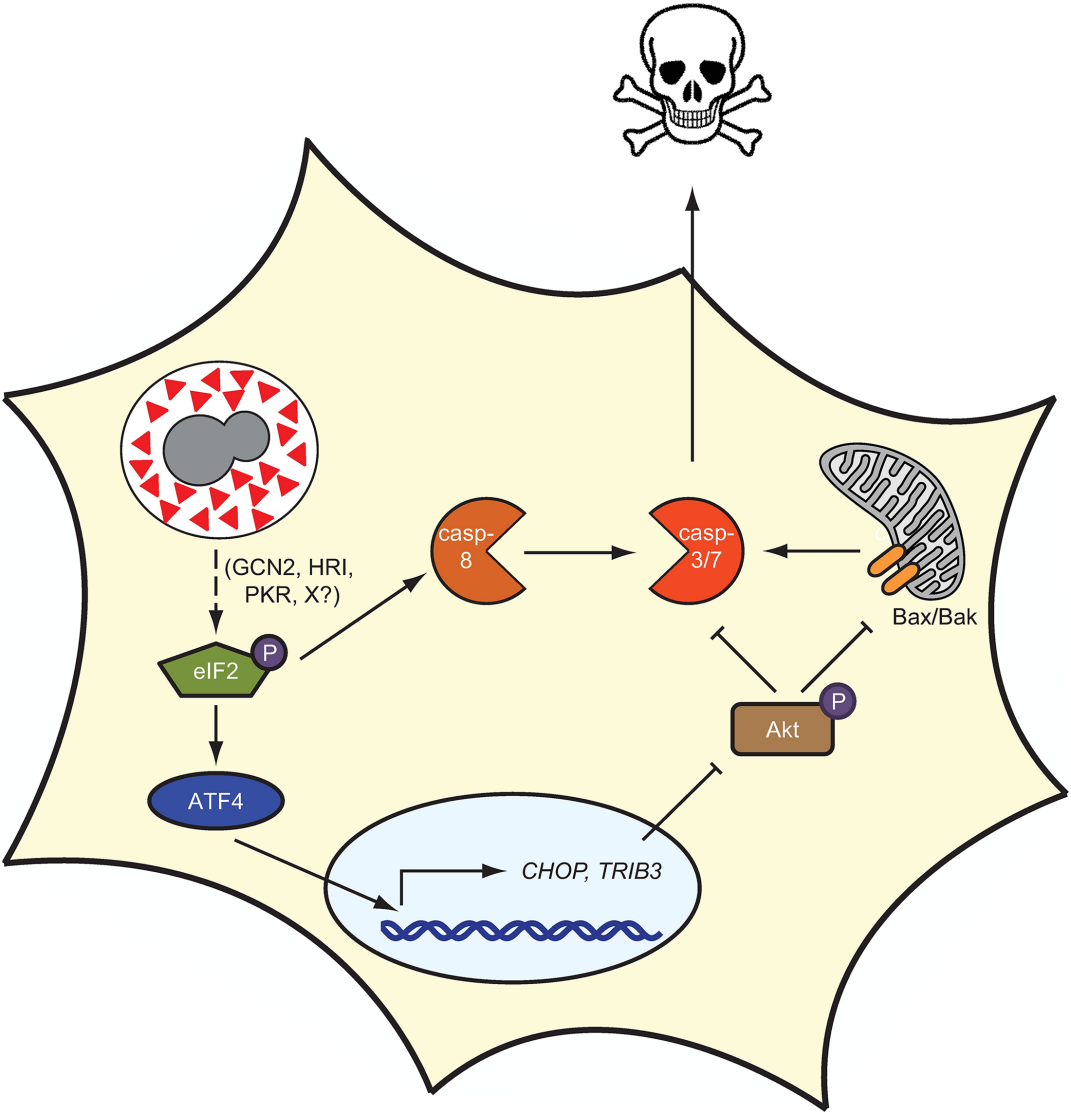
Model of Cbp1-mediated host-cell death during *Hc* infection. During infection, *Hc* yeast produce a large amount of the secreted protein Cbp1 (red triangle). Cbp1 induces phosphorylation of the mammalian protein eIF2α through an unknown mechanism. The increase in phospho-eIF2 leads to the preferential translation of the transcription factor ATF4, which leads to the expression of *CHOP* and *TRIB3*. The pseudokinase Tribbles 3 inhibits Akt phosphorylation, thereby promoting Bax/Bak oligomerization, ultimately resulting in caspase-3/7 activation, which is enhanced by caspase-8 activation. The activation of the executioner caspases ultimately results in macrophage death, allowing for the release of live fungal cells.

In sum, we propose the following model: during *Hc* infection, intracellular yeast produce the secreted protein Cbp1. As the fungus replicates, levels of Cbp1 continue to rise, ultimately resulting in ISR activation and an increase in the levels of *CHOP*. *CHOP* in turn induces expression of *TRIB3*, which triggers a decrease in phospho-Akt levels. This decrease in phospho-Akt leads to an increase in the activity of several pro-apoptotic BH3-only proteins [55], which promote Bax/Bak oligomerization in the outer mitochondrial membrane. As we showed previously, Bax/Bak are required for optimal susceptibility of macrophages to *Hc*-mediated killing [6]. Bax/Bak oligomerization, in addition to the increase in caspase-8 activity stimulated by *Hc* infection, leads to the activation of the executioner caspases-3/7, ultimately resulting in apoptosis of the infected cell and the release of live yeast that are able to colonize new host cells in subsequent rounds of infection (Fig 9). One intriguing facet of this model is that Cbp1 acts as a molecular timer of host-cell lysis: the ISR is triggered only when a threshold level of Cbp1 is attained, thus allowing sufficient fungal replication to occur before the demise of the host cell.

Finally, the discovery that Cbp1 activates the ISR implies that Cbp1 represents a new category of *Hc* virulence factors. Of the handful of known *Hc* virulence factors [56], the others identified thus far promote pathogenesis by enabling *Hc* yeast to survive in the potentially hostile environment of the host. Examples include a siderophore used for iron acquisition within the classically iron-limited environment of the host cell [57], as well as catalases [58] and a secreted superoxide dismutase [59] necessary for combatting reactive oxygen species. Based on our observations, we propose that one function of Cbp1 is to promote pathogenesis by activating a host signaling cascade that ultimately results in macrophage death, allowing for escape of live *Hc* yeast from host cells and efficient spread of the pathogen throughout the host organism.

## Materials and methods

### Culture conditions

Bone marrow-derived macrophages (BMDMs) from 6 to 8 week old female mice were isolated from femurs and tibias and maintained in BMM (bone marrow macrophage media) as described previously [57]. J774.1 cells (ATCC) were maintained in Dulbecco’s modified Eagle’s medium (DMEM) high glucose (UCSF Cell Culture Facility) with 10% Fetal Bovine Serum (FBS; Hyclone or Gibco), penicillin and streptomycin (UCSF Cell Culture Facility). U937 cells (ATCC) were maintained in RPMI medium 1640 (Gibco) with 10% heat-inactivated FBS (Hyclone or Gibco), 2 mM glutamine (UCSF Cell Culture Facility), 110 μg/mL sodium pyruvate (UCSF Cell Culture Facility), penicillin (UCSF Cell Culture Facility), and streptomycin (UCSF Cell Culture Facility). U937 cells were differentiated with 100 nM phorbol 12-myristate 13-acetate (PMA; UCSF Cell Culture Facility). *H*. *capsulatum* cultures were grown in liquid *Histoplasma* macrophage medium (HMM) using an orbital shaker or on HMM agarose plates [60]. All cells were maintained at 37°C with 5% CO_2_. GSK2606414 (PERK inhibitor I; EMD Millipore) and tunicamycin (Santa Cruz Biotechnology) were dissolved in DMSO.

### *Hc* strains

*H*. *capsulatum* strain G217B *ura5Δ* (WU15) was a kind gift from William Goldman (University of North Carolina, Chapel Hill). For all studies involving the *cbp1* mutant, “wildtype” refers to G217 *ura5Δ* transformed with a *URA5*-containing episomal vector (pLH211), *cbp1* refers to G217B*ura5Δcbp1*::*T-DNA* as previously described [6] transformed with the same URA5-containing episomal vector, and “complemented” strain refers to G217B*ura5Δcbp1*::*T-DNA* transformed with the *URA5*-containing episomal plasmid bearing the wild-type *CBP1* gene (pDTI22) as previously described [6].

Primers for alanine scanning mutagenesis of *CBP1* were designed using PrimerX (http://www.bioinformatics.org/primerx/). Primer sequences are included in supplemental material (S2 Table). Mutations were introduced into pDTI22 [6] with site-directed mutagenesis using standard cloning techniques. The fusion gene consisting of the sequence encoding the G217B Cbp1 signal peptide and the sequence encoding the mature Cbp1 from *P*. *brasiliensis* strain Pb03 was synthesized by Genewiz, Inc. The fusion gene was then cloned into pDTI22, replacing G217B *CBP1* while leaving the flanking sequences unchanged.

For all Cbp1 constructs, approximately 25ng of PacI-linearized DNA was electroporated into the *cbp1* mutant and the G217B*ura5Δ* parental strain [6] as previously described [61]. The plasmid pLH211 was used as the vector control. Transformants were selected on HMM agarose plates.

### Cbp1 protein alignment

The genomic sequences of known *CBP1* homologs are consistent with a conserved two-intron gene structure, but the associated protein predictions are often inconsistent with this conserved splicing pattern (e.g., *Emmonsia crescens* KKZ65414.1) or protein predictions are not available (e.g., *Histoplasma capsulatum* Tmu GCA_000313325.1). Therefore, protein sequences were inferred by TBLASTN [62] alignment of the *Histoplasma capsulatum* G186AR protein sequence (AAC39354.1) to the genome assemblies of *Histoplasma capsulatum* G217B (GCA_000170615.1), WU24 (NAm1, GCA_000149585.1), H88 (GCA_000151005.2), H143 (GCA_000151035.1), and Tmu (GCA_000313325.1); *Paracoccidioides lutzii* Pb01 (GCA_000150705.2); *Paracoccidioides brasiliensis* Pb03 (GCA_000150475.2) and Pb18 (GCA_000150735.2); and *Emmonsia crescens* UAMH 3008 (GCA_001008285.1). The inferred protein sequences were aligned with PROBCONS [63], and alignments were formatted with JALVIEW [64].

### Detection of Cbp1 in culture supernatants

Four-day *Hc* cultures were collected and yeast were pelleted by centrifugation. The supernatants were sterile-filtered through 0.22μm filters, and the filtrates were concentrated using Amicon Ultra Centrifugal Filter Units with a 3 kDa cutoff (EMD Millipore). Protein concentration was quantified using the Bio-Rad protein assay (Bio-Rad Laboratories). Equal amounts of protein were separated by SDS-PAGE, and proteins were visualized by staining the gel with Coomassie Brilliant Blue G-250 (Fisher BioReagents).

### Macrophage infections

Macrophage infections with *Hc* strains in the G217B background were performed as described previously [6,57]. Briefly, the day before infection, macrophages were seeded in tissue culture-treated dishes. On the day of infection, yeast cells from logarithmic-phase *Hc* cultures (OD_600_ = 5–7) were collected, resuspended in the appropriate macrophage media, sonicated for 3 seconds on setting 2 using a Fisher Scientific Sonic Dismembrator Model 100, and counted using a hemacytometer. Infection with G186AR required a slightly different protocol due to the tendency of this strain to form large aggregates of cells. G186AR yeast cells from a logarithmic-phase culture were sonicated for 30 1-second pulses, vortexed for 30 seconds, sonicated for 30 1-second pulses, and then vortexed for another 30 seconds to disrupt clumps of yeast cells. The cells were then pelleted, resuspended in the appropriate media, sonicated for 30 1-second pulses, and vortexed for 30 seconds. The cells were then spun at low speed (400 rpm) to pellet large yeast aggregates. The remaining cells in the supernatant, which were single cells or very small aggregates of two or three yeast, were then counted using a hemacytometer. Depending on the multiplicity of infection (MOI), the appropriate number of yeast cells was then added to the macrophages. After a 2-hour phagocytosis period, the macrophages were washed once with PBS and then fresh media was added. For infections lasting longer than 2 days, fresh media was added to the cells approximately 48 hpi.

U937 cells were differentiated with 100 nM PMA three days prior to infection. One day prior to infection, adherent cells were collected by scraping, counted with a hemacytometer, and seeded at a density of 4x10^5^ cells per well in a 24-well plate. The following day, the cells were infected in quadruplicate as described above, although the phagocytosis period was extended to 12 hours to accommodate the reduced phagocytosis rate of differentiated U937 cells for *Hc* cells.

### Cytotoxicity assays

To screen strains expressing mutant alleles of *CBP1*, J774.1 cells were seeded (3.75 x 10^4^ cells per well of a 24-well plate) and infected as described above in triplicate wells per time point. Three independent transformants per alanine mutant were tested. Each day for 4 days after infection, macrophage monolayers were washed once with PBS, fixed and stained with methylene blue staining solution (0.2% methylene blue [Sigma Aldrich], 20% ethanol) at room temperature for 15 min, washed 3 times with PBS, and then imaged.

To quantify macrophage lysis, BMDMs were seeded (7.5 x 10^4^ cells per well of a 48-well plate) and infected as described above. At the indicated time points, the amount of LDH in the supernatant was measured as described previously [65]. BMDM lysis is calculated as the percentage of total LDH from uninfected macrophages lysed in 1% Triton-X at the time of infection. Due to continued replication of BMDMs over the course of the experiment, the total LDH at later time points is greater than the total LDH from the initial time point, resulting in an apparent lysis that is greater than 100%.

### Intracellular replication

BMDMs were seeded (7.5 x 10^4^ cells per well of a 48-well plate) and infected in triplicate as described above. At the indicated time points, culture supernatants were removed and 250 μl of ddH2O was added. After incubating at room temperature for 10 min, the macrophages were mechanically lysed by vigorous pipetting. The lysate was collected, sonicated to disperse any clumps, counted, and plated on HMM agarose in appropriate dilutions. After incubation at 37°C with 5% CO_2_ for 12–14 days, CFUs were enumerated. To prevent any extracellular replication from confounding the results, intracellular replication was not monitored after the onset of macrophage lysis.

### Transwell dissemination assay

The day before infection, 1x10^7^ wildtype or *CHOP*^*-/-*^ BMDMs were seeded in 15 cm tissue culture dishes. The following day, the cells were infected with wildtype *Hc* at an MOI of 2 or mock infected as described above. The same day, wildtype BMDMs were also seeded in 6-well plates at a density of 7.5x10^5^ cells per well. Approximately 24 hours after infection, the wildtype and *CHOP*^*-/-*^ BMDMs from the 15 cm plates were collected by scraping, and viable cells, as determined by trypan blue exclusion, were counted using a hemacytometer. These BMDMs were then seeded on the underside of 0.4 μm polyester transwells (Costar) at a density of 4x10^5^ cells per transwell in 1 mL BMM. After 2 hours, excess liquid was aspirated, and the transwells were inverted over the wildtype BMDMs in 6-well plates. Approximately 18 hours later, when the infected transwell BMDMs had begun to lyse but the lower macrophage monolayer was still intact, the transwells were removed, and the lower BMDMs were washed once with PBS and fresh BMM was added. CFUs and cytoxicity were measured as described above.

### RNA isolation and RT-PCR

For RNA isolation from cultured cells, BMDMs were seeded (1x 10^6^ cells per well of a 6-well plate) and infected in triplicate as described. Triplicate wells of infected macrophages were lysed in 1 mL total of QIAzol (Qiagen). U937 cells were seeded at 4x10^5^ cells per well of a 24-well plate and infected in quadruplicate as described. Quadruplicate wells were lysed in 1 mL total of QIAzol (Qiagen). Lung samples were thawed on ice, and 500 μL of homogenate was pelleted at 4°C and resuspended in 1 mL of QIAzol (Qiagen). After addition of chloroform, total RNA was isolated from the aqueous phase using Econo-spin columns (Epoch Life Science) and then subjected to on-column PureLink DNase (Invitrogen) digestion. To generate cDNA, 2–4 μg total RNA was reverse transcribed using Maxima Reverse Transcriptase (Thermo Scientific), an oligo-dT primer, and pdN9 primers following manufacturer’s instructions. Splice isoforms of Xbp1 were detected by performing non-quantitative PCR on 1:10 dilutions of cDNA using Phusion High-Fidelity DNA polymerase (New England BioLabs) with the following cycling conditions: 98°C for 30s, followed by 35 cycles of 98°C (10 s), 65°C (30 s), and 72°C (30 s), followed by 72°C for 10 min. The resulting amplicons were separated and visualized on a 2.5% agarose gel containing ethidium bromide. Quantitative PCR was performed on 1:10 to 1:50 dilutions of cDNA template using FastStart SYBR Green MasterMix with Rox (Roche). Reactions were run on an Mx3000P machine (Stratagene) and analyzed using MxPro software (Stratagene). Cycling parameters were as follows: 95°C for 10 min, followed by 40 cycles of 95°C (30 s), 55°C (60 s), and 72°C (30 s), followed by dissociation curve analysis. Abundances of *ERdj4*, *SEL1L*, *BiP*, *CHOP*, and *TRIB3* were normalized to *HPRT* levels. Primer sequences are listed in S2 Table.

### Protein isolation and Western blots

For protein isolation, BMDMs were seeded (1x 10^6^ cells per well of a 6-well plate) and infected in triplicate as describe. Triplicate wells of macrophages were lysed in a 300 μl total of radioimmunoprecipitation assay (RIPA) buffer (50 mM TrisHCl pH8, 150 mM NaCl, 1% NP-40, 0.5% sodium deoxycholate, 0.1% SDS) with Halt Protease and Phosphatase Inhibitor Cocktail (Thermo Scientific). Insoluble debris was removed by centrifugation. Protein concentrations were determined using Pierce BCA Protein Assay Kit (Thermo Scientific). Equivalent amounts of protein were separated by SDS-PAGE and transferred to nitrocellulose. Membranes were incubated with antibodies per manufacturer’s suggestions. Blots were imaged on an Odyssey CLx and analyzed using ImageStudio2.1 (Licor). The following primary antibodies were used: phospho-PERK (ThermoFisher Scientific G.305.4), phospho-eIF2α (Cell Signaling Technology 9721), α-tubulin (Santa Cruz Biotechnology YOL1/24; sc-53030), ATF4 (Cell Signaling Technology D4B8; 11815), CHOP (Cell Signaling Technology L633F7; 2895), TRIB3 (Calbiochem ST1032), phospho-Akt (Cell Signaling Technology C313E5E; 2965), Akt (Cell Signaling Technology 40D4; 2920). Images were processed with Adobe Photoshop, which was utilized on occasion to change the order of lanes in the image to group like samples (e.g. lytic strains or non-lytic strains) together.

### *In vitro* caspase activity

BMDMs were seeded at 2.5 x 10^4^ cells per well in solid white 96-well plates and infected as described above. Caspase-3/7 and caspase-8 activities were measured using Caspase-Glo 3/7 Assay and Caspase-Glo 8 Assay, respectively, according to manufacturer’s instructions (Promega). GSK2606414 (EMD Millipore) was dissolved in DMSO and added to macrophages after the 2-hour phagocytosis period at a final concentration of 3 μM where indicated. Luminescence was measured on a Wallac Victor2 microplate reader (PerkinElmer).

### Mouse infections

Eight-to-twelve week-old female C57Bl/6 (Jackson Laboratory stock 000664) or *CHOP*^-/-^ (B6.129S(Cg)-*Ddit3*^tm2.1Dron^/J; Jackson Laboratory stock 005530) mice were anesthetized with isoflurane and infected intranasally with wildtype *Hc* yeast. The inoculum was prepared by collecting mid-logarithmic phase (OD_600_ = 5–7) yeast cultures, washing once with PBS, sonicating for 3 seconds on setting 2 using a Fisher Scientific Sonic Dismembrator Model 100, counting with a hemacytometer, and diluting in PBS so that the final inoculum was approximately 25 μl. To monitor survival, animals were infected with 1x10^6^ yeast per mouse. To monitor *in vivo* colonization, animals were infected with 3x10^5^ yeast per mouse. Infected mice were monitored daily for symptoms of disease, including weight loss, hunching, panting, and lack of grooming. For survival curve analysis, mice were euthanized after they exhibited 3 days of sustained weight loss greater than 25% of their maximum weight in addition to one other symptom. For *in vivo* colonization, five mice per genotype were sacrificed at the indicated time points, and lungs and spleens were harvested. The organs were homogenized in PBS and plated on brain heart infusion (BHI) agar plates supplemented with 10% sheep’s blood (Colorado Serum Company). After plating, the remaining organ homogenate was snap frozen in liquid nitrogen and stored at -80°C. CFUs were enumerated after 10–12 days of growth at 30°C.

### Flow cytometry

For flow cytometry analysis, mice were sacrificed with avertin, and lungs were perfused with PBS and then removed. Lungs were then dissociated in Hanks Buffered Salt Solution (HBSS) containing 0.5 mg DNase I (Roche) per mouse, and 0.75 U Collagenase P (Roche) per mouse using a GentleMACS Tissue Dissociator (Milteny Biotec). Red blood cells were hypotonically lysed with ACK lysis buffer and the remaining cells were filtered through a 70 μM cell strainer (BD Biosciences.) 2x10^6^ lung cells were resuspended in FACS buffer (PBS containing 1% heat-inactivated FBS, 1 mM EDTA, 10 μg/mL CD16/32, and 0.1% sodium azide) and stained with CellEvent Caspase-3/7 Green Detection Reagent (Invitrogen) according to manufacturer’s instructions. The cells were then washed with FACS buffer and stained with Fixable Viability Dye eFluor 450 (eBiosciences) for 20 minutes. Cells were then incubated with CD16/32 (BioLegend) for 20 minutes, then stained for 30 minutes with the appropriate antibodies, fixed in BD Stabilizing Fix (BD Biosciences), and stored at 4°C until analysis on an LSR II (BD Biosciences). Antibodies used to identify alveolar macrophages were as follows: BV650-CD45 (BioLegend), PE-SiglecF (BD Biosciences), BV605-CD11b (BioLegend), Alexa700-CD11c (BioLegend), and BV711-F4/80 (BioLegend). Flow cytometry data were analyzed using FlowJo version 10. Apoptotic cells were defined as Fixable Viability Dye eFluor 450^+^ and Caspase-3/7 Green Detection Reagent^+^ double-positive cells.

### Statistical analysis

Two-tailed t-tests were performed using Excel (Microsoft). Two-tailed Mann-Whitney tests and statistical analysis for mouse survival and colonization experiments were performed using Prism (GraphPad Software) as described in the figure legend.

### Ethics statement

All mouse experiments were performed in compliance with the National Institutes of Health *Guide for the Care and Use of Laboratory Animals* and were approved by the Institutional Animal Care and Use Committee at the University of California San Francisco (protocol AN155431-01). Mice were euthanized by CO2 narcosis and cervical dislocation consistent with American Veterinary Medical Association guidelines.

## Supporting information

S1 Fig Alanine scan of Cbp1 generates secreted proteins with a range of abilities to lyse macrophages during *Hc* infection. Alanine scanning mutagenesis was performed on mature Cbp1, and the resulting alleles were expressed in our *cbp1* null *Hc* strain. **(A)** Mutants were initially screened for secretion into *Hc* yeast culture supernatants, with at least four transformants per allele analyzed. In this representative image, 2 μg/μl of total protein from culture supernatants from four-day old cultures of wildtype *Hc*, *cbp1*, *cbp1*+*cbp1*^*P56A*^, *cbp1*+*cbp1*^*L57A*^, or *cbp1*+*cbp1*^*D58A*^ was separated by SDS-PAGE. Proteins were visualized in the gel by Coomassie staining. Cbp1 is the prominent band at approximately 8 kDa, as determined by mass spectrometry. Mass spectrometry was also used to identify the prominent 19 kDa band seen in the supernatant of the *cbp1* mutant strain as YPS-3, a secreted protein produced by *Hc* yeast that contains a chitin-binding domain (S1 References). YPS-3 is sometimes observed prominently in supernatants from 4-day old cultures. **(B)** Mutant alleles of Cbp1 that were secreted from *Hc* were qualitatively assessed for their ability to lyse macrophages during *Hc* infection. At least three transformants per mutant were analyzed. In this representative image, J774.1 cells, a murine macrophage-like cell line, were mock infected (uninf) or infected with wildtype *Hc*, *cbp1*, *cbp1*+*cbp1*^*I55A*^, *cbp1*+*cbp1*^*D58A*^, or *cbp1*+*cbp1*^*T66A*^ at an MOI of 10 in duplicate wells. Macrophage lysis was visualized at 4 dpi by staining the cell monolayer with methylene blue.
(TIF)

S2 Fig *Hc* strains secreting different Cbp1 variants grow to high levels within macrophages. **(A)** 5 mL of culture supernatants from 3-day old cultures of the indicated *Hc* strains were concentrated to 250 μL. Equivalent volumes were then separated by SDS-PAGE, and proteins were visualized by Coomassie staining. **(B)** BMDMs were infected with the indicated *Hc* strains at an MOI of 5. At the indicated time points, *Hc* CFUs were enumerated to monitor intracellular fungal burden. To insure that CFUs reflected intracellular but not extracellular yeast replication, CFUs were not measured after the onset of macrophage lysis. Each value is an average of triplicate wells ± standard deviation.
(TIF)

S3 Fig Alignment of mature Cbp1 sequences. Mature Cbp1 sequences from 6 *Hc* strains, 2 *Pb* strains, 1 *Paracoccidioides lutzii* (*Pl*) strain, and 1 *Emmonsia crescens* (*Ec*) strain were inferred and aligned as described in Materials and Methods. *Emmonsia* spp. are emerging dimorphic fungal pathogens (53), and the role of Cbp1 in their pathogenesis has yet to be explored. Arrows show the location of the two alanine mutants used in this study. Colors correspond to amino acid properties.
(TIF)

S4 Fig The mammalian unfolded protein response (UPR). The mammalian UPR consists of three sensor proteins that detect ER stress: IRE1, ATF6, and PERK. Upon activation, IRE1 oligomerizes and autophosphorylates, stimulating its RNase activity. Activated IRE1 splices out a non-canonical intron from the *Xbp1u* transcript, resulting in *Xbp1s*. Xbp1s is a transcription factor that controls the expression of many genes, including *ERdj4* and *BiP*. Upon activation, ATF6 is cleaved into ATF6n, which is a transcription factor that promotes the expression of genes such as *SEL1L* and *BiP*. Upon activation, PERK oligomerizes and autophosphorylates, then phosphorylates eIF2α, leading to a stress response through selective translation.
(TIF)

S5 Fig Lytic *CBP1* alleles lead to CHOP and TRIB3 production in infected macrophages. BMDMs were treated with 2.5 μg/mL tunicamycin (Tm), infected with indicated *Hc* strains at an MOI of 5, or mock infected (uninf). CHOP and TRIB3 protein levels were assessed by Western blots at 12 hpi, with α-tubulin as the loading control.
(TIF)

S6 Fig Robust *TRIB3* expression during *Hc* infection is dependent on *CHOP*. Wildtype or *CHOP*^-/-^ BMDMs were mock infected (uninf) or infected with the indicated *Hc* strains at an MOI of 5. *TRIB3* expression was assessed 12 hpi by RT-qPCR, with expression values normalized to uninfected wildtype BMDMs.
(TIF)

S7 Fig *CHOP* and *TRIB3* induction precedes macrophage death in a variety of infections. **(A)** BMDMs were infected with the G186AR *Hc* strain at an MOI of 5 or mock infected (uninf). **(B)** Differentiated U937 cells were mock infected (uninf) or infected with the indicated *Hc* strains at an MOI of 5. Macrophage death was measured by LDH release. Relative abundances of *CHOP* and *TRIB3* transcripts were assessed by RT-qPCR at 12 hpi and normalized to uninfected macrophages.
(TIF)

S8 Fig Wildtype *Hc* has no growth defect in *CHOP*^-/-^ or *TRIB3*^-/-^ BMDMs. Wildtype, *CHOP*^*-/-*^, and *TRIB3*^*-/-*^ BMDMs were infected with wildtype *Hc* at an MOI of 1, and intracellular fungal burdens were assessed by CFUs at the indicated time points. Each value is an average of triplicate wells ± standard deviation.
(TIF)

S9 Fig *CHOP*^*-/-*^ mice are resistant to *Hc* infection. **(A)** Wildtype and *CHOP*^*-/-*^ mice (n = 5) were infected with 1 x 10^6^ mCherry-producing *Hc* yeast. The percentage of infected (mCherry^+^) CD45^+^ cells was determined by flow cytometry of lungs collected 3 dpi. **(B)** Wildtype and *CHOP*^*-/-*^ mice (n = 5) were infected with 3x10^5^ *Hc* yeast. Lungs were collected and homogenized at 1 dpi, RNA was isolated from half of the homogenate, and *TRIB3* expression was assessed by RT-qPCR. **p<0.01, ANOVA. **(C)** Wildtype and *CHOP*^*-/-*^ mice (n = 11) were mock infected (uninf) or infected with 1 x 10^6^ wildtype *Hc* yeast, and animal weights were monitored daily. Animals were sacrificed if they met the euthanasia criteria described in the materials and methods.
(TIF)

S1 References Citations referenced in supporting material. (DOCX)

S1 Table Summary of Cbp1 alanine scan results. (XLSX)

S2 Table Primers used in this study. (XLSX)
