# Supplementary figures and images for: The transcription factor CHOP, an effector of the integrated stress response, is required for host sensitivity to the fungal intracellular pathogen Histoplasma capsulatum
Source: PLoS Pathog. 2017 Sep 27;13(9):e1006589. doi: 10.1371/journal.ppat.1006589 (PMC5633207; doi:10.1371/journal.ppat.1006589)

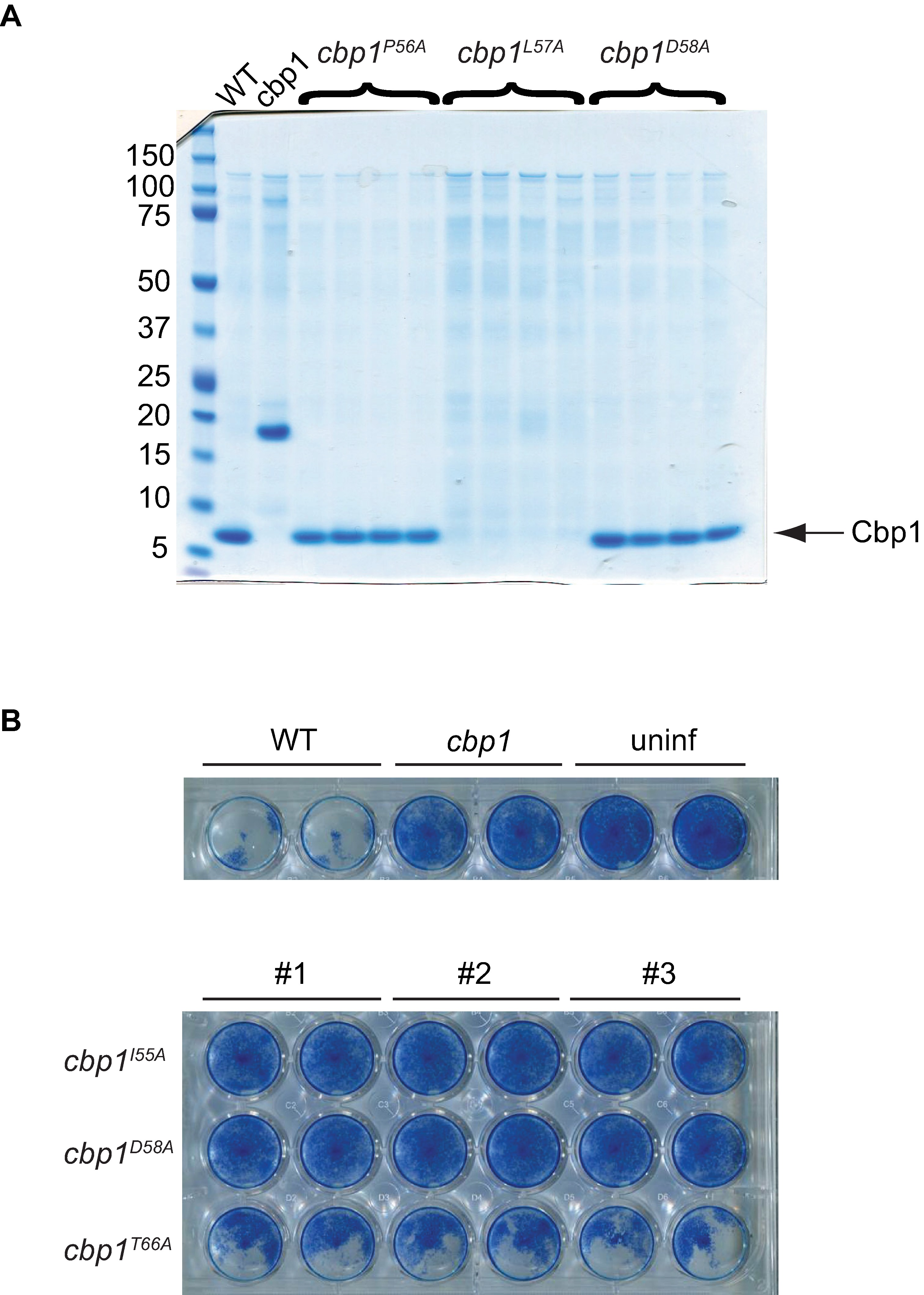

Supplement: S1 Fig — Alanine scanning mutagenesis was performed on mature Cbp1, and the resulting alleles were expressed in our cbp1 null Hc strain. (A) Mutants were initially screened for secretion into Hc yeast culture supernatants, with at least four transformants per allele analyzed. In this representative image, 2 μg/μl of total protein from culture supernatants from four-day old cultures of wildtype Hc, cbp1, cbp1+cbp1P56A, cbp1+cbp1L57A, or cbp1+cbp1D58A was separated by SDS-PAGE. Proteins were visualized in the gel by Coomassie staining. Cbp1 is the prominent band at approximately 8 kDa, as determined by mass spectrometry. Mass spectrometry was also used to identify the prominent 19 kDa band seen in the supernatant of the cbp1 mutant strain as YPS-3, a secreted protein produced by Hc yeast that contains a chitin-binding domain (S1 References). YPS-3 is sometimes observed prominently in supernatants from 4-day old cultures. (B) Mutant alleles of Cbp1 that were secreted from Hc were qualitatively assessed for their ability to lyse macrophages during Hc infection. At least three transformants per mutant were analyzed. In this representative image, J774.1 cells, a murine macrophage-like cell line, were mock infected (uninf) or infected with wildtype Hc, cbp1, cbp1+cbp1I55A, cbp1+cbp1D58A, or cbp1+cbp1T66A at an MOI of 10 in duplicate wells. Macrophage lysis was visualized at 4 dpi by staining the cell monolayer with methylene blue. (TIF) [file ppat.1006589.s001.tif]

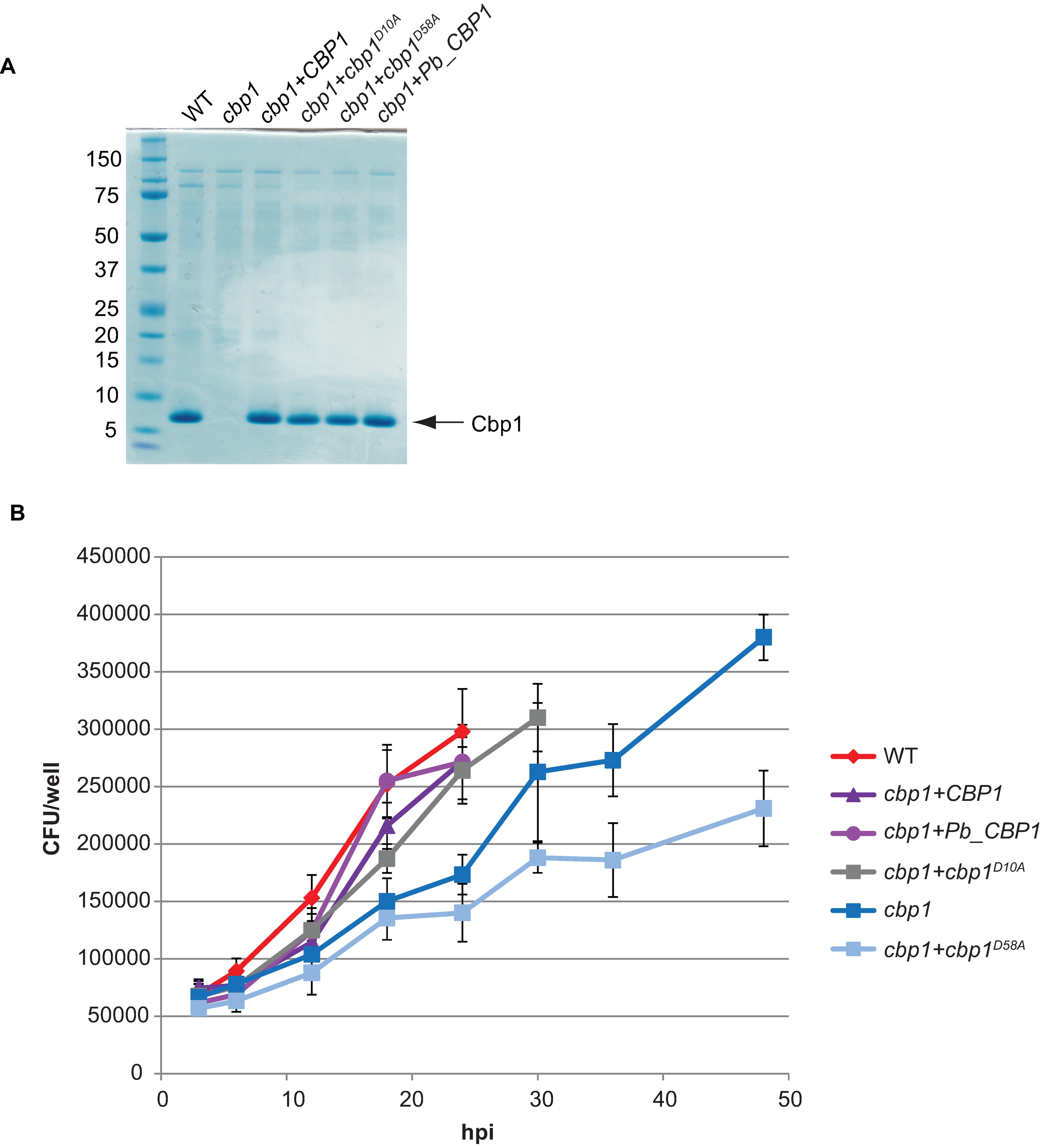

Supplement: S2 Fig — (A) 5 mL of culture supernatants from 3-day old cultures of the indicated Hc strains were concentrated to 250 μL. Equivalent volumes were then separated by SDS-PAGE, and proteins were visualized by Coomassie staining. (B) BMDMs were infected with the indicated Hc strains at an MOI of 5. At the indicated time points, Hc CFUs were enumerated to monitor intracellular fungal burden. To insure that CFUs reflected intracellular but not extracellular yeast replication, CFUs were not measured after the onset of macrophage lysis. Each value is an average of triplicate wells ± standard deviation. (TIF) [file ppat.1006589.s002.tif]

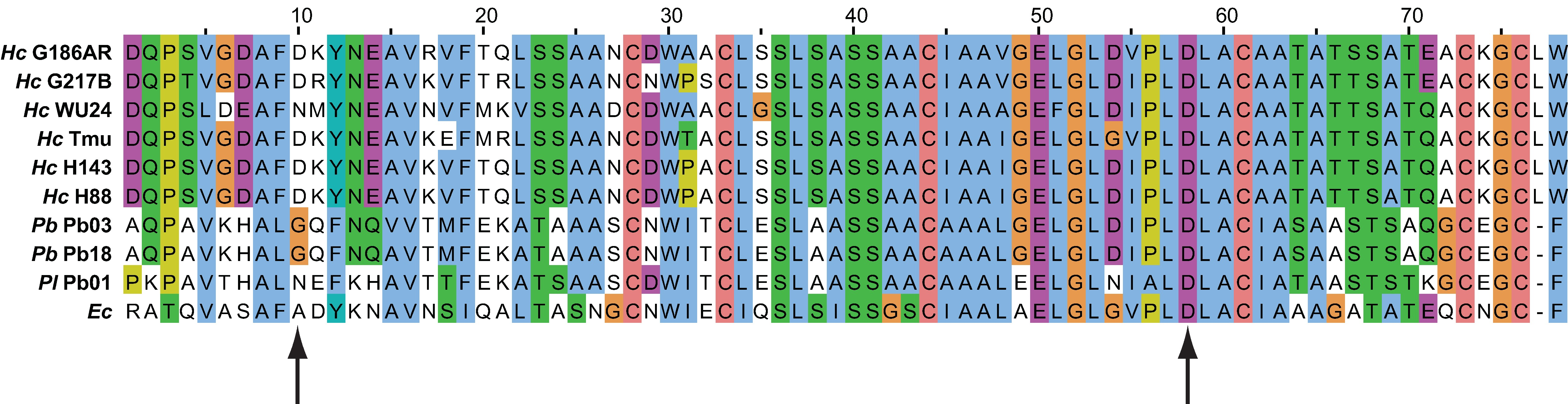

Supplement: S3 Fig — Mature Cbp1 sequences from 6 Hc strains, 2 Pb strains, 1 Paracoccidioides lutzii (Pl) strain, and 1 Emmonsia crescens (Ec) strain were inferred and aligned as described in Materials and Methods. Emmonsia spp. are emerging dimorphic fungal pathogens (53), and the role of Cbp1 in their pathogenesis has yet to be explored. Arrows show the location of the two alanine mutants used in this study. Colors correspond to amino acid properties. (TIF) [file ppat.1006589.s003.tif]

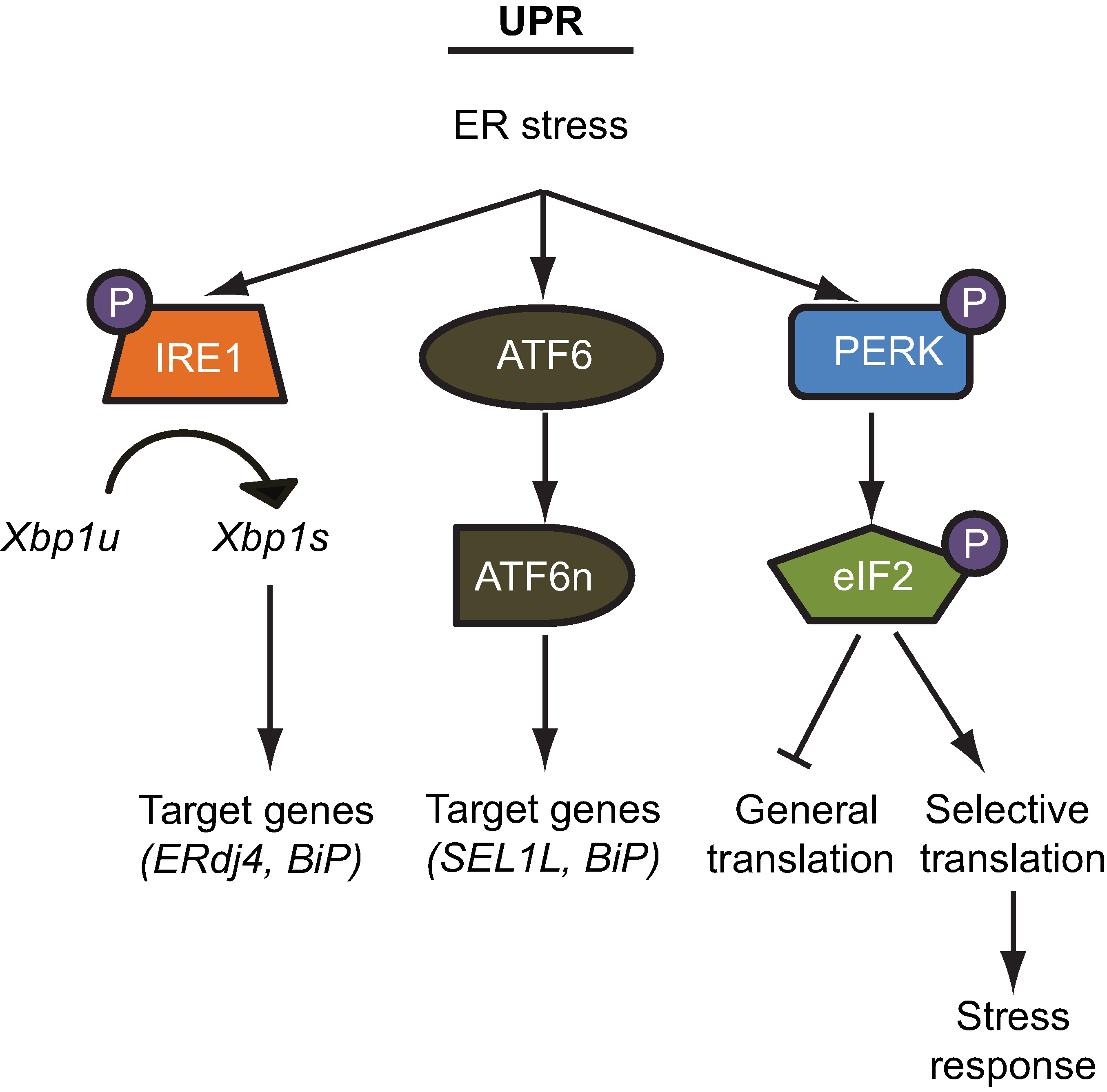

Supplement: S4 Fig — The mammalian UPR consists of three sensor proteins that detect ER stress: IRE1, ATF6, and PERK. Upon activation, IRE1 oligomerizes and autophosphorylates, stimulating its RNase activity. Activated IRE1 splices out a non-canonical intron from the Xbp1u transcript, resulting in Xbp1s. Xbp1s is a transcription factor that controls the expression of many genes, including ERdj4 and BiP. Upon activation, ATF6 is cleaved into ATF6n, which is a transcription factor that promotes the expression of genes such as SEL1L and BiP. Upon activation, PERK oligomerizes and autophosphorylates, then phosphorylates eIF2α, leading to a stress response through selective translation. (TIF) [file ppat.1006589.s004.tif]

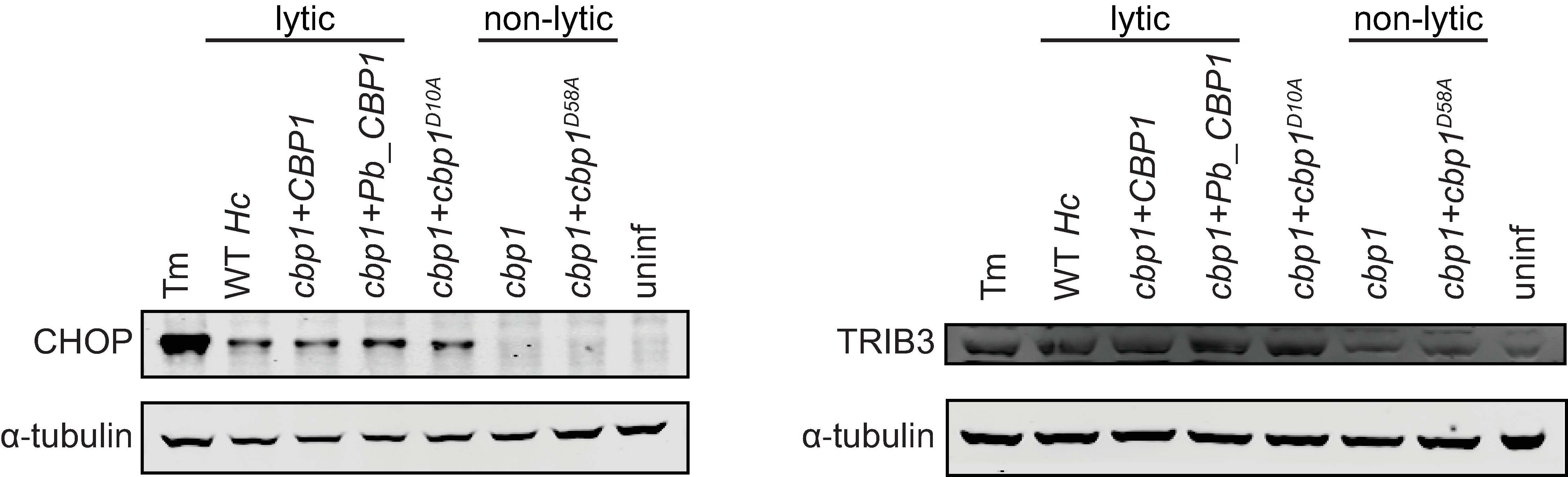

Supplement: S5 Fig — BMDMs were treated with 2.5 μg/mL tunicamycin (Tm), infected with indicated Hc strains at an MOI of 5, or mock infected (uninf). CHOP and TRIB3 protein levels were assessed by Western blots at 12 hpi, with α-tubulin as the loading control. (TIF) [file ppat.1006589.s005.tif]

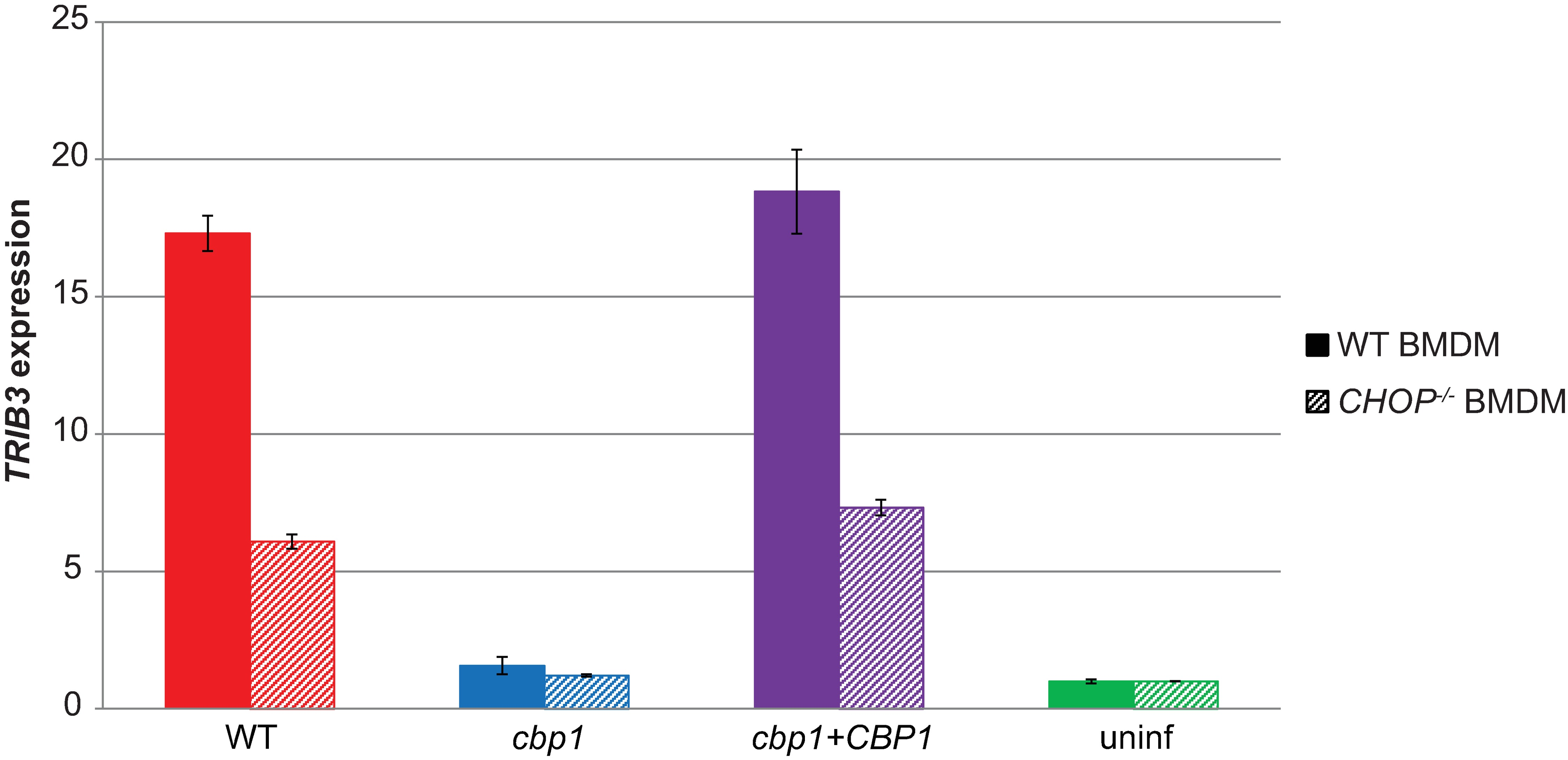

Supplement: S6 Fig — Wildtype or CHOP-/- BMDMs were mock infected (uninf) or infected with the indicated Hc strains at an MOI of 5. TRIB3 expression was assessed 12 hpi by RT-qPCR, with expression values normalized to uninfected wildtype BMDMs. (TIF) [file ppat.1006589.s006.tif]

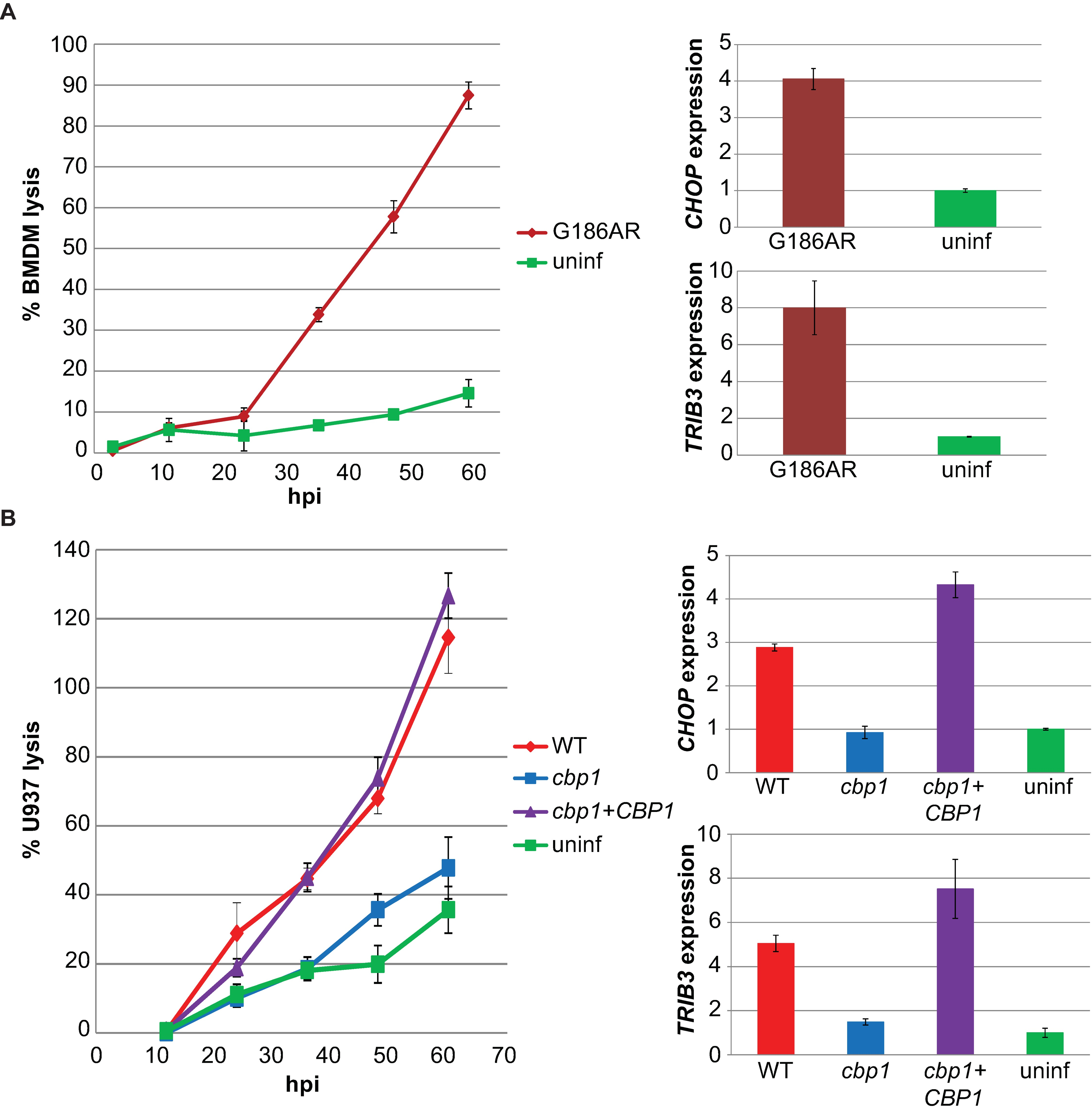

Supplement: S7 Fig — (A) BMDMs were infected with the G186AR Hc strain at an MOI of 5 or mock infected (uninf). (B) Differentiated U937 cells were mock infected (uninf) or infected with the indicated Hc strains at an MOI of 5. Macrophage death was measured by LDH release. Relative abundances of CHOP and TRIB3 transcripts were assessed by RT-qPCR at 12 hpi and normalized to uninfected macrophages. (TIF) [file ppat.1006589.s007.tif]

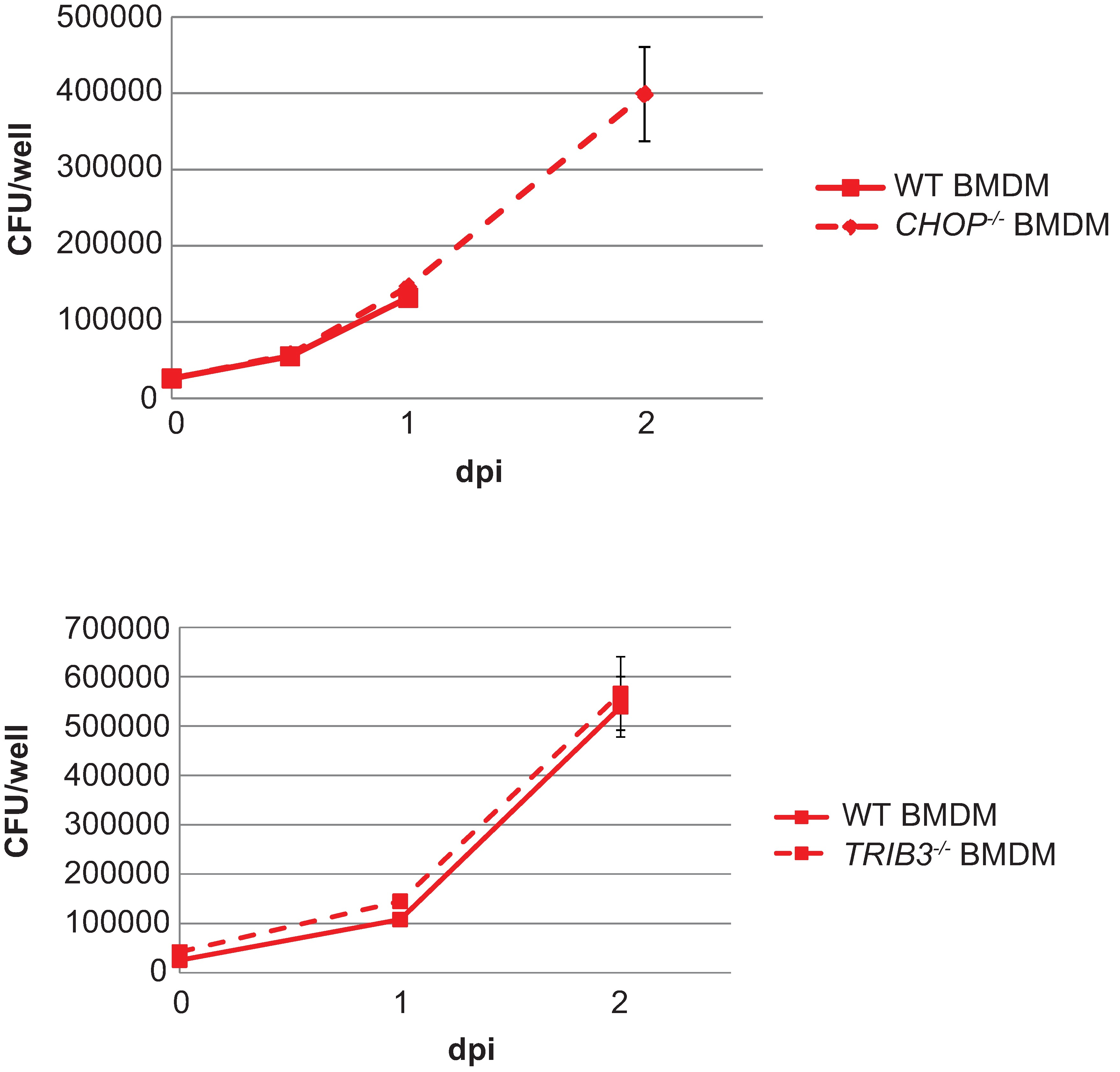

Supplement: S8 Fig — Wildtype, CHOP-/-, and TRIB3-/- BMDMs were infected with wildtype Hc at an MOI of 1, and intracellular fungal burdens were assessed by CFUs at the indicated time points. Each value is an average of triplicate wells ± standard deviation. (TIF) [file ppat.1006589.s008.tif]

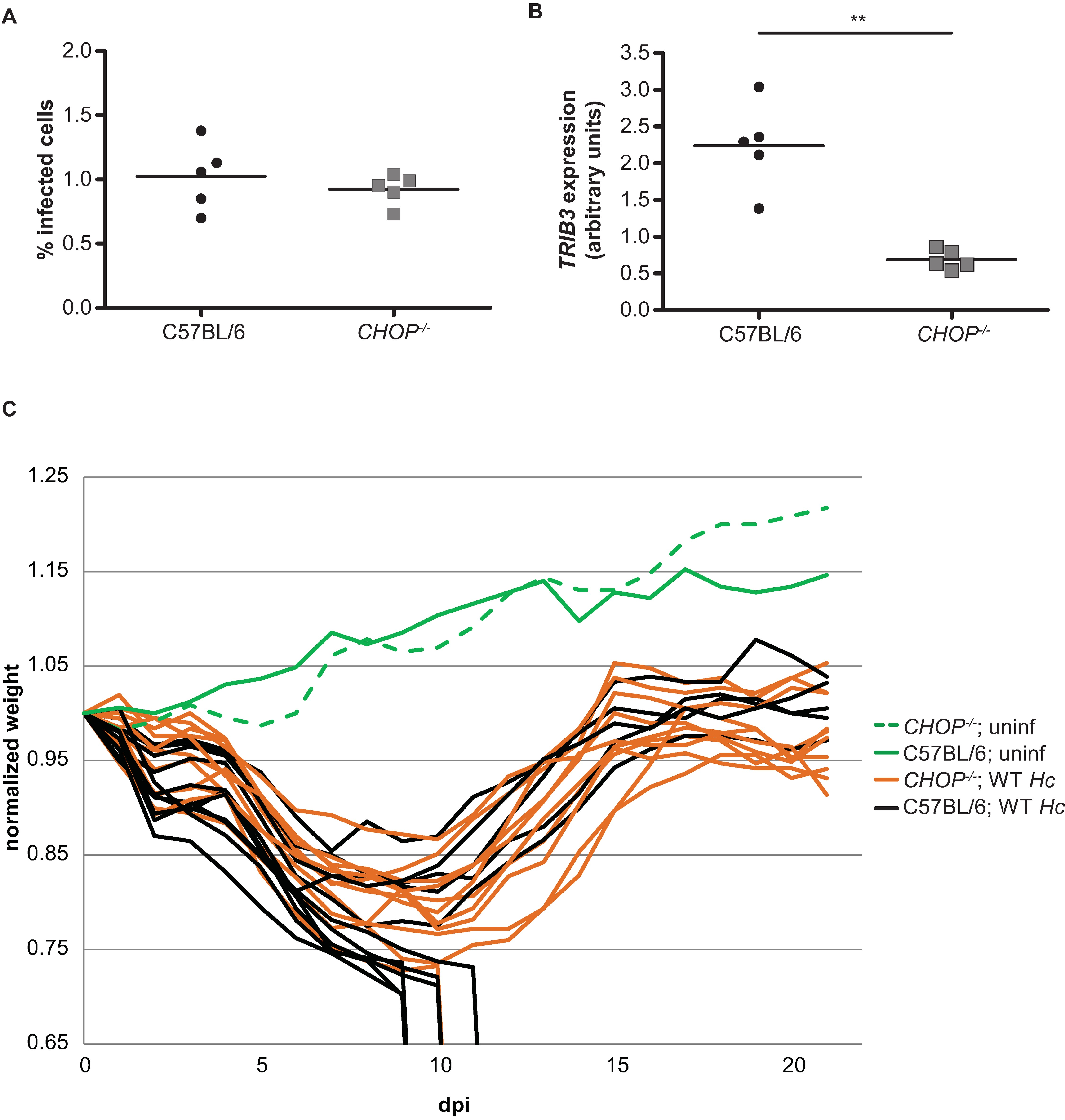

Supplement: S9 Fig — (A) Wildtype and CHOP-/- mice (n = 5) were infected with 1 x 10^6^ mCherry-producing Hc yeast. The percentage of infected (mCherry+) CD45+ cells was determined by flow cytometry of lungs collected 3 dpi. (B) Wildtype and CHOP-/- mice (n = 5) were infected with 3x10^5^ Hc yeast. Lungs were collected and homogenized at 1 dpi, RNA was isolated from half of the homogenate, and TRIB3 expression was assessed by RT-qPCR. **p<0.01, ANOVA. (C) Wildtype and CHOP-/- mice (n = 11) were mock infected (uninf) or infected with 1 x 10^6^ wildtype Hc yeast, and animal weights were monitored daily. Animals were sacrificed if they met the euthanasia criteria described in the materials and methods. (TIF) [file ppat.1006589.s009.tif]
