# Supplementary material for: The transcription factor CHOP, an effector of the integrated stress response, is required for host sensitivity to the fungal intracellular pathogen Histoplasma capsulatum
Source: PLoS Pathog. 2017 Sep 27;13(9):e1006589. doi: 10.1371/journal.ppat.1006589 (PMC5633207; doi:10.1371/journal.ppat.1006589)
Supplement: S1 References — (DOCX) [file ppat.1006589.s010.docx]

**Supporting References**

1. Bohse ML, Woods JP. Surface localization of the Yps3p protein of Histoplasma capsulatum. Eukaryot Cell. 2005;4(4):685–93.
